# Supplementary material for: Livogena: The Ikteros Curse—A Jaundice Narrative Card and Board Game for Medical Students
Source: MedEdPORTAL. 2024 Feb 6;20:11381. doi: 10.15766/mep_2374-8265.11381 (PMC10844581; doi:10.15766/mep_2374-8265.11381)

# Game Cards

# Note: This appendix should be printed as cards and placed in a deck. The deck should be shuffled before the game. Students shall draw one card from the check when each sub-team plays. The answered cards should be kept separately, and unanswered cards can be inserted into the deck and shuffled again

# 1

Name the by-product of heme degradation whose deficiency causes microcytic hypochromic anemia

# 2

Which reaction utilizes NADPH in heme degradation pathway?

# 3

What are the effects of CO released during the degradation of heme

to biliverdin?

# 4

What is the end product of heme degradation in birds, amphibians, and rabbits?

# 5

Mention the oxidation state of the mineral released as a by-product of heme degradation

# 6

Name the transport protein for, lipophilic bilirubin formed in the reticulo-endothelial cells

# 7

Name any three drugs that can displace bilirubin from its transport protein?

# 8

Name the cytosolic proteins to which bilirubin binds once it enters

the liver cells

# 9

Mention the other function of Ligandin

# 10

**Common treatment for neonatal hyperbilirubinemia when bilirubinlevels rise above 15mg/dL**

# 11

Which class of CNS depressants can be used to improve the symptoms of jaundice in

Crigler-Najjar syndrome?

# 12

How can mild jaundice be avoided in Gilbert disease without drug supplementation?

# 13

What is the threshold value of bilirubin in blood above which it is known as hyperbilirubinemia?

# 14

At what level does bilirubin diffuse into tissues producing yellow discoloration of sclera, conjunctiva, skin, and mucous membranes?

# 15

What is the result of Van den Berg test

at pH 5 for hydrophilic bilirubin?

# 16

**What is the result of Van den Berg test in blood with indirect**

**bilirubin – 0.2-0.6 mg/dL and direct bilirubin 0-0.2 mg/dL?**

# 17

What is the reagent that is used in the test that measures urinary urobilinogen?

# 18

How is the liver function test in Dubin-Johnson syndrome?

# 19

**Give the expected laboratory findings of blood-free bilirubin in pre-hepatic, hepatic and post-hepatic jaundice**

# 20

**Give the expected laboratory findings of blood-conjugated bilirubin in pre-hepatic, hepatic and post-hepatic jaundice**

# 21

Which isomer of coproporphyrin is increased in

Dubin-Johnson syndrome?

# 22

Give the level of unconjugated bilirubin in blood in Crigler Najjar syndrome 1 & 2

# 23

Sensorineural hearing loss is a serious and permanent effect in which complication of jaundice?

# 24

Conjugated hyperbilirubinemia, yellow discoloration of the bulbar conjunctiva, clay-colored stool and high levels of CA 19-9 are likely to suggest

# 25

Yellowing of eyes and skin is also a common complication of which condition associated with increased accumulation of a substance required for heme synthesis, normal growth, and development.


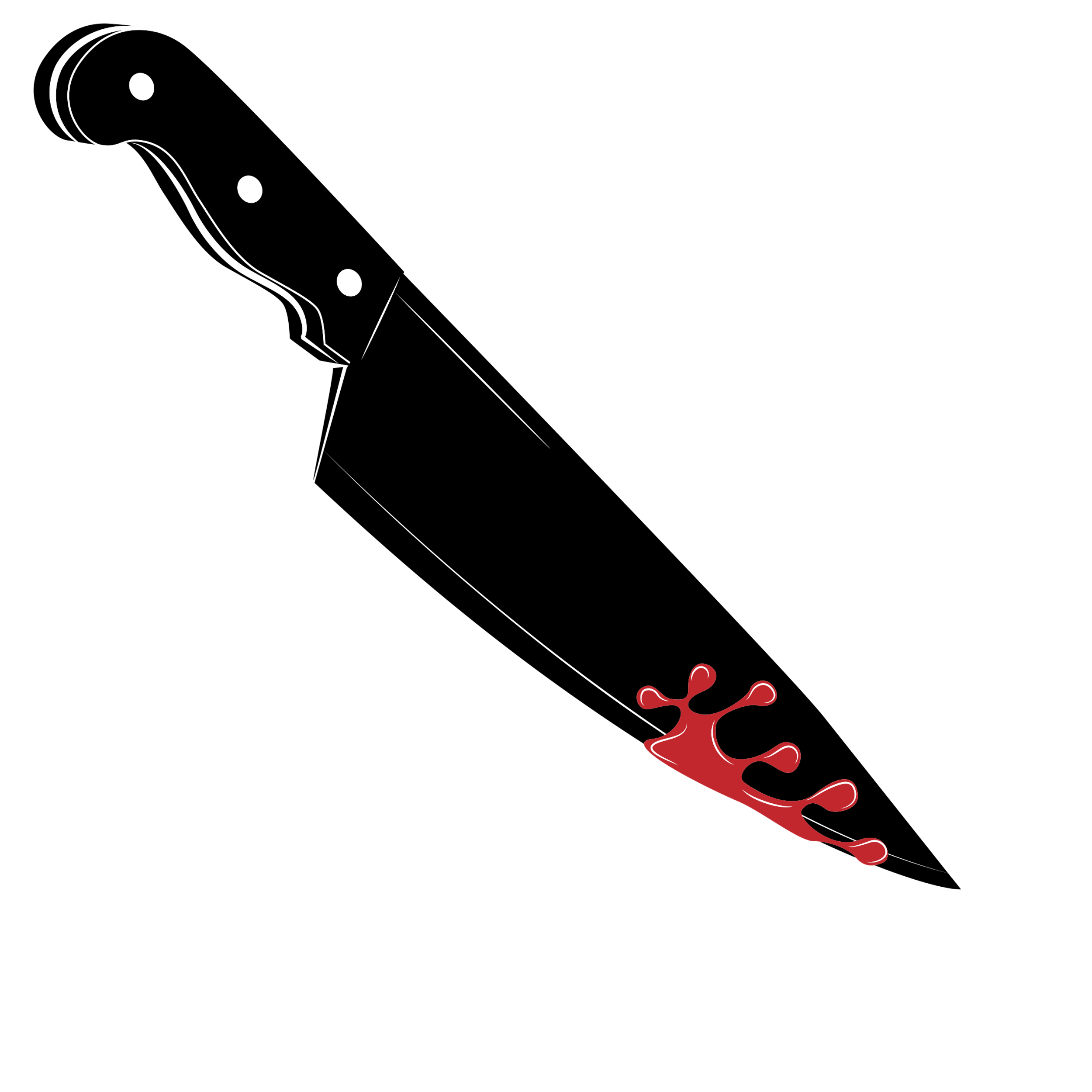


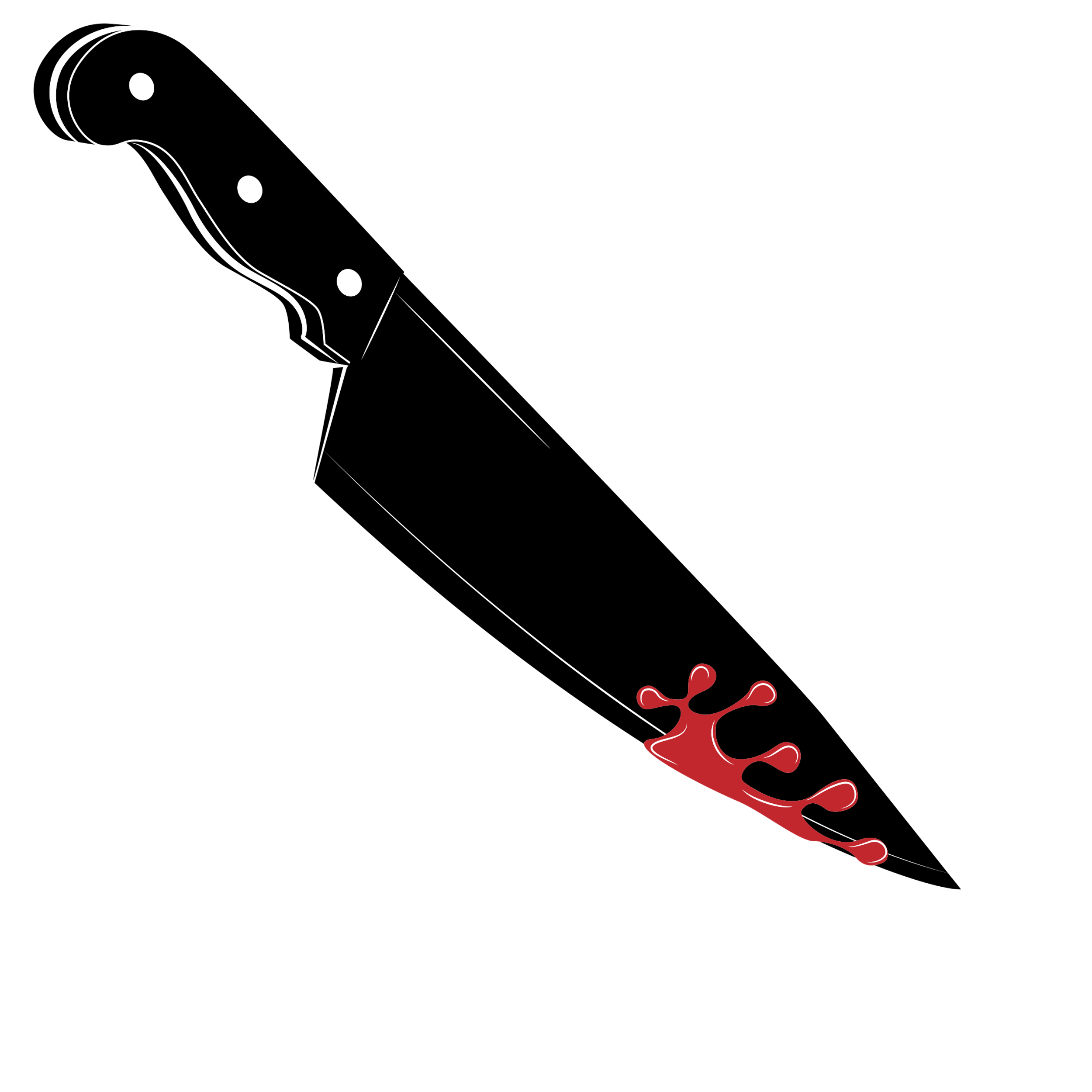


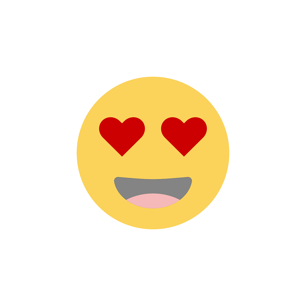


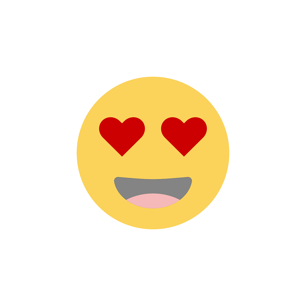


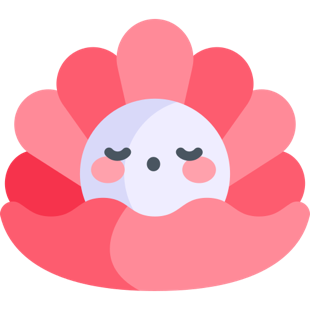


# 1

Which reaction of monosaccharide is involved in the production of uronic acid utilized in the conjugation of insoluble compounds to soluble products?

# 2

Name any three inhibitors of UDP glucuronyl transferase that interferes in the process of conjugation and may produce jaundice.

# 3

Which is the rate limiting step in the catabolism of heme?

# 4

**Name the ATP-dependent transporter of conjugated bilirubin that is responsible for excretion of bilirubin into bile and give its location**

# 5

What is the function of intestinal bacteria on direct bilirubin?

# 6

About 20% of UBG formed in the intestine is returned to the liver and a small fraction is also excreted in the urine as a result of

# 7

Prolonged administration of antibiotics causes green tinged feces especially in children. Give reason

# 8

How can you assess the effectiveness of biliary drainage?

# 9

What is the treatment of choice for a child born with severe hemolytic disease

/ Erythroblastosis fetalis?

# 10

What is the route of administration of Rh immunoglobulins in a Rh-negative mother?

# 11

**What would be the suggested action in a TB patient who develops non-hemolytic hyperbilirubinemia**

**due to intake of Rifampicin?**

# 12

Which test is used to measure the compounds that impart color to urine and feces?

# 13

What is Hay's test?

And give the principle of the test

# 14

Name the two tests that measure urinary bilirubin

# 15

How can you differentiate physiological and pathological

jaundice with the time of onset of symptoms in a newborn infant?

# 16

How does specific gravity of urine gets altered in hepatic jaundice?

# 17

**Give the expected laboratory findings of serum Alkaline Phosphatase in pre-hepatic, hepatic and post-hepatic jaundice**

# 18

**Give the expected laboratory findings of Urine- bile salts in pre-hepatic, hepatic and post-hepatic jaundice**

# 19

In which type of jaundice Fouchet's test results are negative

# 20

What is the level of serum ALT, AST & ALP in

Gilbert syndrome?

# 21

In chronic hepatocellular disorders, how is prothrombin time altered?

# 22

How does plasma albumin level change in hemolytic jaundice which results in increased production of unconjugated bilirubin?

# 23

Mention other non-hepatic causes of rise in urine bilirubin (direct)

# 24

Which vitamin intake may cause false-negative results in urine- conjugated bilirubin detection?

# 25

Presence of covalently bound conjugated bilirubin in blood

but the absence of conjugated bilirubin in urine may be due to which condition?


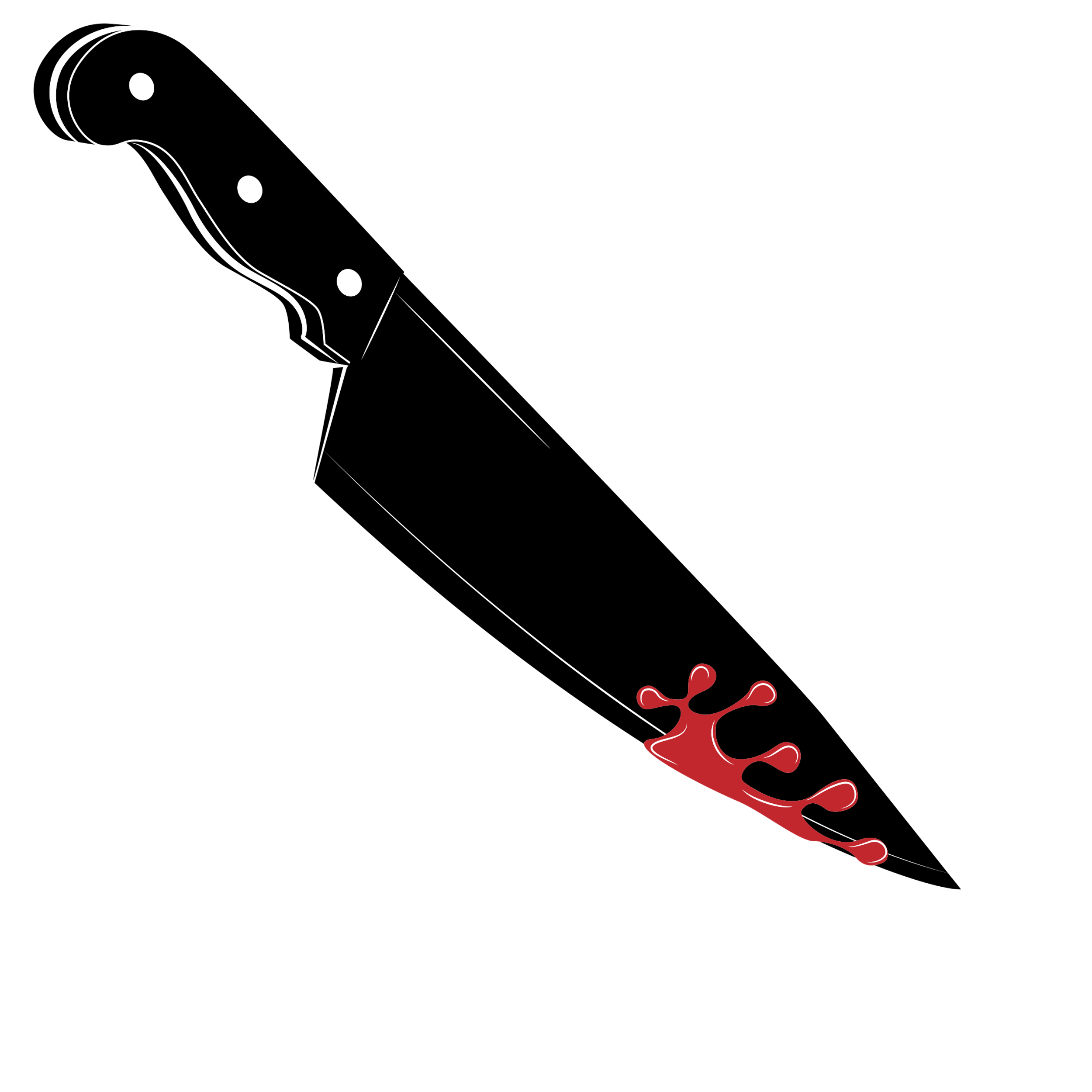


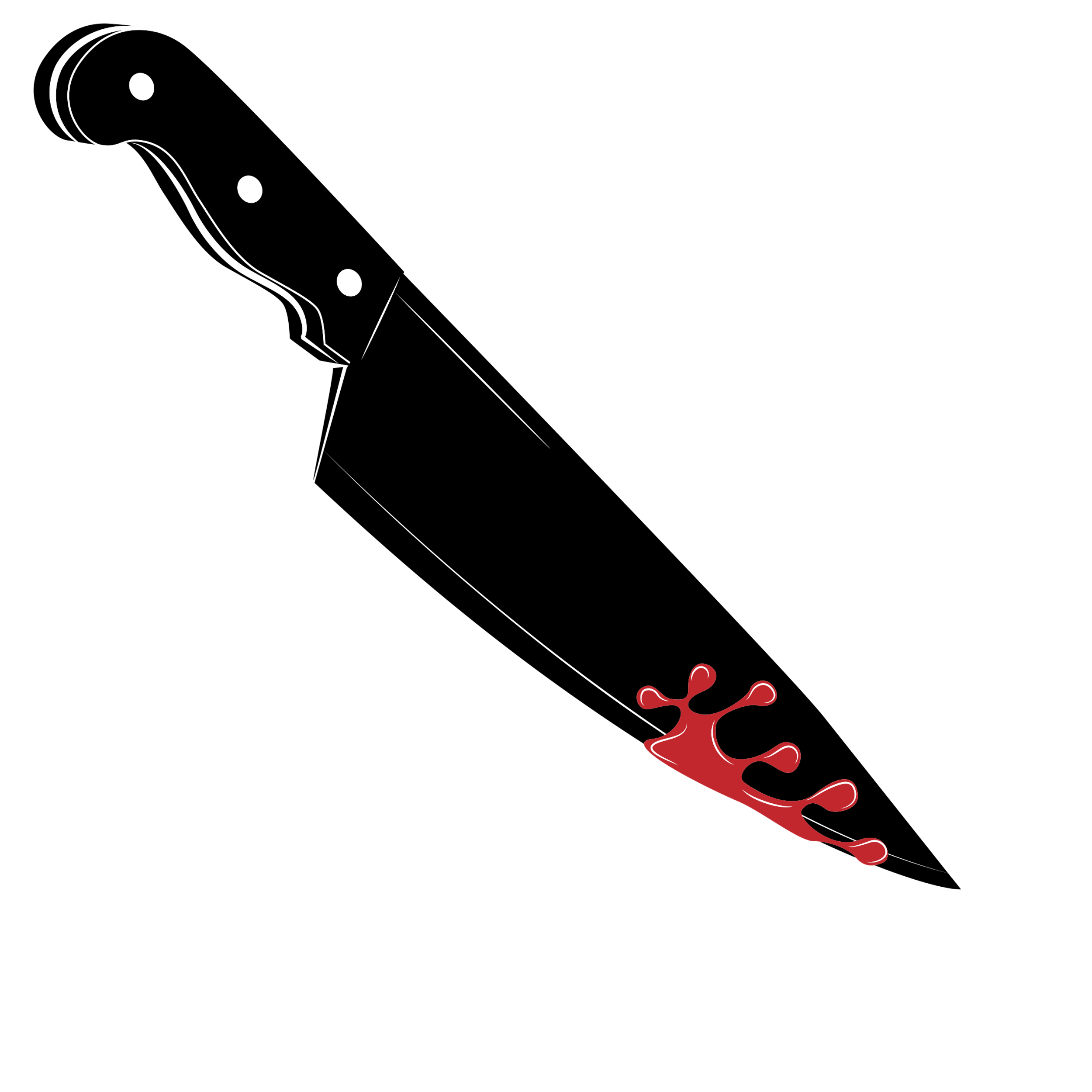


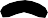

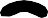

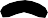

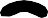


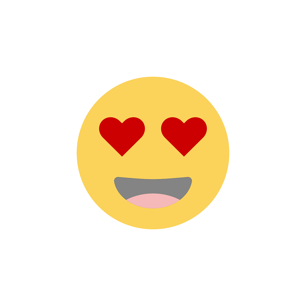


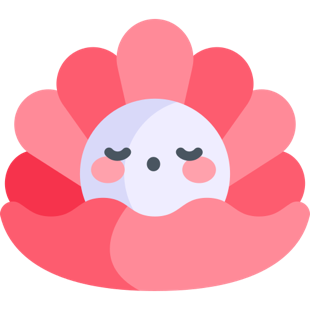


# 1

What is the metabolic defect in Black Liver Jaundice?

# 2

What are the non-pathological conditions that can cause mild jaundice in Gilbert disease?

# 3

Mention a cause for non-hemolytic increase of unconjugated bilirubin in blood

# 4

High level of estrogen derivative in fetal blood causes prolongation of jaundice in breastfed infant. True or false

# 5

Absence of bilirubinuria is seen in?

# 6

What is the effect of bilirubin in brain mitochondria?

# 6

Enlarged lymph glands of porta hepatis may be a sign of obstruction leading to jaundice in which condition?

# 8

**What is the mode of inheritance of**

**Dubin-Johnson syndrome?**

# 9

What is the drug of choice that is given to reduce pruritis in jaundice?

# 10

Administration of phenobarbital reduces blood level of bilirubin and is beneficial in which type of Crigler-Najjar syndrome?

# 11

What is the definitive treatment of Acalculous cholecystitis? – a form of cholecystitis that results in impaired gallbladder emptying leading to obstructive jaundice

# 12

What are the dietary items that should be avoided in jaundice?

# 13

Give the normal value of serum Alkaline Phosphatase

# 14

Give the normal value of SGOT & SGPT

# 15

Name the other serum marker that raises during hemolysis and give its normal value

# 16

Give the normal level of total cholesterol in blood

# 17

What is MRCP in radiology?

# 18

Give the expected laboratory findings of Urine- conjugated

bilirubin in pre-hepatic, hepatic and post-hepatic jaundice

# 19

Give the expected laboratory findings

of Urine urobilinogens in pre-hepatic, hepatic

and post-hepatic jaundice

# 20

Give the expected laboratory findings of fecal urobilinogen in pre-hepatic, hepatic

and post-hepatic jaundice

# 21

Is Delta bilirubin seen in urine of patients with jaundice and normal

glomerular function? Why?

# 22

**What are the serological investigations that you would order to check for acute hepatitis B infection in a patient**

**who presents with jaundice?**

# 23

What is the first sign of recovery that is noticed

in patients with obstructive jaundice?

# 24

Van den Berg test is indirect positive, this suggests that the patient may be suffering from which type of jaundice?

# 25

What is the first impaired activity in hepatocellular jaundice and what is the finding in diazotized sulfanilic acid test?


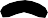

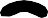

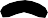

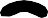


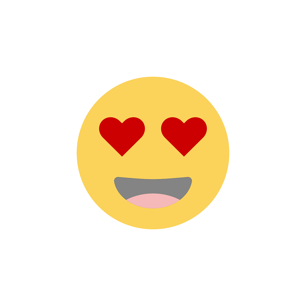


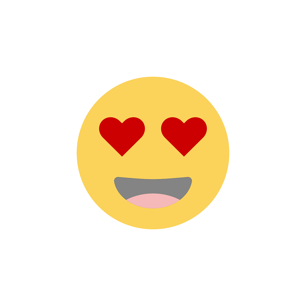


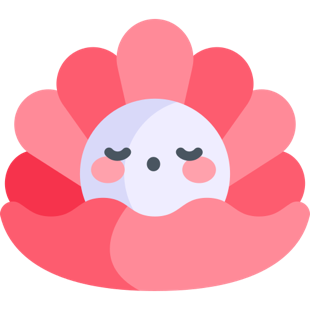

Supplement: Supplementary file 1 — Pre- and Posttest.docGame Instructions.docGame Story.docGame Video.mp4Game Board.jpgGame Cards.docxQuestions and Answer Key.docGame Activities.docPerceptions Questionnaire.docFeedback Questionnaire.doc [file mep_2374-8265.11381-s001.zip › F. Game Cards.docx]
