# Supplementary material for: Livogena: The Ikteros Curse—A Jaundice Narrative Card and Board Game for Medical Students
Source: MedEdPORTAL. 2024 Feb 6;20:11381. doi: 10.15766/mep_2374-8265.11381 (PMC10844581; doi:10.15766/mep_2374-8265.11381)
Supplement: Supplementary file 1 — Pre- and Posttest.docGame Instructions.docGame Story.docGame Video.mp4Game Board.jpgGame Cards.docxQuestions and Answer Key.docGame Activities.docPerceptions Questionnaire.docFeedback Questionnaire.doc [file mep_2374-8265.11381-s001.zip › _Educational Summary Report_11381.pdf]

MedEdPORTAL® The AAMC Journal of Teaching and Learning Resources

Original Publication Open Access

Livogena: The Ikteros Curse—A Jaundice Narrative Card and Board Game for Medical Students

Krishna Mohan Surapaneni, PhD, MHPE*

*Corresponding author: krishnamohan.surapaneni@gmail.com

Abstract

Introduction: Jaundice is a common condition that requires integrating knowledge of biochemistry, physiology, pathology, and general medicine. However, medical students face difficulty in learning with passive teaching methods. To enhance their learning, an educational story game that promotes active learning and assessment with immediate feedback was implemented. **Methods:** This jaundice game was named Livogena: The Ikteros Curse—denoting the liver as the principal organ and jaundice (icterus) as a problem. One hundred fifty first-year medical students were divided into small groups to play using a game board and cards. The players moved ahead on the game board by providing the correct answer and completing the activities. The first team to reach the end was the winner. Perceptions and feedback questionnaires were distributed to students at the end of the game. Individual views about the game were recorded for qualitative analysis. Also, to analyze the effectiveness of this intervention, pre- and posttests on jaundice were conducted. **Results:** Livogena: The Ikteros Curse resulted in a highly significant improvement in students' knowledge and application skills in jaundice, from 5.5 ($SD = 2.4$) in the pretest to 11.2 ($SD = 7.6$) in the posttest for 20 marks ($p < .001$). Students perceived and rated the game exceptionally positively. **Discussion:** This educational game significantly increased learners' understanding of the concepts of jaundice. Highly positive perceptions from students further affirm this to be a creative innovation to enhance their learning and application of knowledge in an active and team-based learning environment.

Keywords
Board Game, Gamification, Jaundice, Narrative, Story, Biochemistry & Cell Biology, Case-Based Learning, Games

Educational Objectives

By the end of this activity, learners will be able to:

1. Identify different types of jaundice with causes, mechanisms, and clinical features.
2. Interpret the laboratory findings in each type of jaundice.
3. Identify the differential diagnoses based on laboratory findings and clinical features.
4. Choose appropriate treatment options for all types of jaundice.

Introduction

Jaundice is a complex metabolic disease that requires a systematic approach for diagnosing the exact underlying pathology and planning effective treatment strategies.[1] Jaundice is introduced in the early medical curriculum and is an important condition for students to learn. However, at that stage, a thorough comprehension of the concepts and proper interpretation of laboratory findings can be a challenge to medical students as they have not yet been exposed to clinics. Also, conventional didactic lectures do not actively involve the students but rather create a passive method of teaching.

Medical education is evolving at a rapid pace, with newer and innovative approaches to teaching-learning methodologies. The introduction of games or game elements in a nongaming context is gaining popularity in terms of creating an immersive and participatory environment for students.[2,3] This pedagogical approach promotes student engagement and reforms medical education into a more diverse and inclusive educational domain.[4] The concept of gamification or game-based learning in medicine has facilitated collaborative learning resulting in higher knowledge retention and improved performance on examinations.[5-7] In addition, the component of storytelling that enables the learner to visualize, process information, and

Citation:
Surapaneni KM. Livogena: The Ikteros Curse–a jaundice narrative card and board game for medical students. *MedEdPORTAL*. 2024;20:11381. https://doi.org/10.15766/mep_2374-8265.11381

Copyright © 2024 Surapaneni. This is an open-access publication distributed under the terms of the Creative Commons Attribution license.

remember content learned by generating such scenes can also be implemented in medical education.[8]

MedEdPORTAL has published numerous innovations in medical education that have promoted active learning and student engagement using cards, boards, and Jeopardy-style games.[9-11] As one step forward, low-fidelity simulations have been integrated into a board game, creating a practical environment for pediatric and emergency medicine students to learn the management of septic shock.[12] Positive results have also been produced by a few resources that utilize the effectiveness of games in learning clinically oriented topics such as anemia,[13] anatomies of the liver and portal venous system,[14] and pathological conditions like cysts or tumors of the liver.[15] However, none of these resources have been constructed in the context of biochemistry and jaundice for medical students.

A targeted needs assessment for our existing curriculum revealed that lack of comprehension and integration were the major reasons why students found the interpretation of case-based questions challenging. Hence, to facilitate their learning, games were identified as an engaging and interesting method to create a sense of exploration in them and promote active learning. To achieve this, Livogena: The Ikteros Curse—an educational story game using cards and boards—was developed.

This game was built upon a story inspired by jaundice to spark interest and motivation to learn through the visualization of characters. It was a card and board style of game in which players were able to recall what they had learned, self-assess their learning strategy, collaborate with others to find the answers, and actively acquire new knowledge through the immediate feedback provided. The game also included tasks, which provided refreshment throughout, rather than being a continuous question-and-answer session. The effectiveness of this innovation was statistically analyzed to check whether there was a significant improvement in the knowledge and interpretation skills of students. Also, perceptions of all types of learners regarding the game were compared to prove that the game facilitated learning for all students regardless of their preferred learning style.

Methods

As per the National Medical Commission of India, in the new competency-based medical education curriculum,[16] beginning from phase I of the bachelor of medicine/bachelor of surgery (MBBS) program, preclinical subjects were integrated with paraclinical and clinical subjects according to structured competencies and specific learning objectives. Jaundice was one of the important conditions integrated into biochemistry, physiology, pathology, and general medicine. Traditionally, these concepts were delivered through didactic lectures or small-group discussions. The Livogena: The Ikteros Curse creative curriculum on jaundice was introduced as a new learning and assessment innovation. *Livogena* denoted the liver, the principal organ in the metabolism of bilirubin; the term *ikteros* was a Greek word for jaundice; and the term *curse* signified an obstacle or challenging condition. This educational story game was implemented with the 150 first-year medical students admitted to the MBBS course for the academic year 2022-2023 at the Panimalar Medical College Hospital & Research Institute, in Chennai, Tamil Nadu, India. As this was a team-based activity, faculty were invited to be facilitators for every participating group. They were informed in a 30-minute orientation session about the game rules and their role as a facilitator in motivating students to actively engage in the game. The game was developed to facilitate learning, assess, and give immediate feedback and was conducted the day after the lecture-based teaching; hence, learners need to have at least baseline knowledge about jaundice to participate in the game.

Before the game, each student was instructed to identify their unique learning style by completing the VARK preferred learning styles questionnaire, version 8.01.[17,18] This was done to compare the effectiveness of the game across different types of learners. However, this was an optional element, and future users need not consider it as essential to reproducing the innovation. Also, the assessment was structured per the first three levels of the Kirkpatrick four levels of evaluation.[19] Hence, students took a pretest with 20 multiple-choice questions (Appendix A) for 20 minutes before the game for later comparison with posttest scores. Of the test's 20 questions, 10 were recall-type questions, and 10 were application-type. Following the pretest, the game instructions (Appendix B) were distributed to the students, who were randomly assigned into 25 small groups with six students in each team. The teams were further subdivided into pairs. Thus, every team had three subteams and one facilitator with a game board and a deck of cards placed in a facedown fashion.

The creative component of the innovation was its development of a story related to jaundice, Livogena: The Ikteros Curse (Appendix C). To motivate the students and instill curiosity in them, this storyline was played as a video (Appendix D) to create an immersive experience beyond the concrete reality of the classroom. The video included the story of the game and the mission to be accomplished by the learners. This preparatory phase for the game took 15 minutes overall.

The game was designed in such a way that it would draw the attention of players and engage them in active discovery. The

Copyright © 2024 Surapaneni. This is an open-access publication distributed under the terms of the Creative Commons Attribution license.

game board consisted of 100 boxes with alternating colors of red, violet, and yellow (Appendix E). A total of nine character boxes were created (Angel Albumina; Spleendora, the unicorn; Galbinos, the evil monsters; newborn Prince Ehrlich; Hepasoteria, the antidote; Witch Verdina; Royal Guards Glucurons; Queen Liverna; and King Rubin) and placed between the color boxes according to the order in which they appeared in the activities. The remaining 91 boxes had 91 corresponding color-matched cards. Out of 91 cards, 75 were cards with questions categorized randomly into knowledge ($n = 25$), differential diagnosis ($n = 25$), investigation ($n = 15$), or treatment ($n = 10$) regarding jaundice. The remaining 16 cards were “fun cards” without any questions but with specific instructions. There were four types of fun cards (Lucky Charm, Hemocide, Kernictears, and Foucheter). Lucky Charm cards gave a bonus move to the players in which they could advance one box forward. With the Hemocide cards, one team could make another team of their choice lose their turn and go back to their previous position. while Kernictears cards caused the playing team to lose their turn and go back to their previous position. Finally, the Foucheter cards allowed the players to save and use them to skip any questions later (Appendix F). The same game materials were used by all the teams. However, the deck of cards was shuffled by the facilitator in front of the students before the start of the game to ensure that the order of questions was randomized for each team.

Each team decided which subteam would play first by drawing a card from the deck. They then took turns answering the questions on the cards to move forward, advancing a color coin along the path with every right answer given by each subteam. The players moved to the color on the board that matched the color on the card. The facilitator had a reference key to check the answers (Appendix G). Moving forward on the board was restricted when a subteam provided no or incorrect answers. The next subteam then continued the game. All the unanswered cards were inserted into the deck and shuffled after every round. The game also included activities when teams landed on character boxes. Bypassing the character boxes was not possible without completing the required activities. These activities were performed by all the members of the team regardless of their subteams. Each activity was given a name, goal, and instructions about the activity that had to be completed. The respective activities were given to the students by the facilitator when they landed on character boxes. The activities were made interesting and relevant to provide refreshment for the learners (Appendix H). The first team to complete all the activities and accomplish the common goal of saving the king was the winner. The estimated time for completion of the game was 60 minutes. At the end of the game, all the answers were discussed with the students by their facilitators in a 30-minute session to ensure complete learning of the content.

Even though it was important that the students also developed an ability to evaluate and use credible resources in their clinical problem-solving, during the game they were not provided with reference ranges for laboratory values. This was done to ensure that the students had strong foundational knowledge and that the game’s competitiveness was maintained. However, educators who are considering reimplementing this game can include a reference key for laboratory values if their students need not remember the reference ranges.

After the game, a posttest was conducted using the same material as the 20-minute pretest to compare the effectiveness of the intervention (Kirkpatrick level 2). In addition, both the retention and application of knowledge gained with this educational approach over a period of time were analyzed by comparing the scores of a high-stakes internal assessment of the intervention batch to previous batches who had not received this innovation (Kirkpatrick level 3). Then, the perceptions and feedback questionnaires were shared online with the students to obtain their views about the game. Perceptions about the game were obtained with a 32-item questionnaire scored on a 5-point Likert scale (Appendix I). Feedback was obtained using an 11-item questionnaire scored on a 10-point scale (Kirkpatrick level 1; Appendix J). Also, in-depth small-group interviews were conducted with each group in which the players and faculty were encouraged to give their views about the game for qualitative analysis.

The means and standard deviations of all continuous variables were descriptively analyzed using univariate statistics, the differences were compared using *t* tests, and analyses of variance, Mann-Whitney *U* tests, and Kruskal-Wallis tests were performed to identify differences in nonnormal distribution. SPSS, version 17 (SPSS Inc.), was used; p values $< .05$ were considered significant. This project was approved by the Institutional Human Ethics Committee of the Panimalar Medical College Hospital & Research Institute (PMCHRI-IHEC-059, dated March 15, 2022).

Results

This innovation was conducted in April 2023 with 150 first-year medical students. All 150 students participated in the game and completed the assessment and evaluation. On comparing the scores of students on the pre- and posttests, the mean score improved significantly from 5.5 ($SD = 2.4$) to 11.2 ($SD = 7.6$) against a 20-point scale ($p < .001$; Kirkpatrick level 2). There

Copyright © 2024 Surapaneni. This is an open-access publication distributed under the terms of the Creative Commons Attribution license.

was equal improvement in both recall and application types of questions. The statistically proven results clearly indicate that the intended educational objectives had been achieved by the end of the game. Assessment of behavior with respect to the retention and application of knowledge over a period of time (Kirkpatrick level 3) was analyzed by comparing the scores of the high-stakes internal assessment. The mean score (out of 100) of the intervention batch improved to 63.8 compared to the previous two batches (50.0 and 54.1), which did not have the game-based intervention, with a p value less than .001.

All the students ($n = 150$, 100% response rate) completed the perceptions and feedback questionnaires distributed at the end of the activity. The mean age of the participants was 18.8 years, with a maximum age of 25 and a minimum age of 18. There was a female preponderance of 76% over 24% for males. Most learners were of the multimodal type (40%), followed by kinesthetic (25%), visual (21%), read/write (11%), and aural learners (3%). Students' perceptions of the game were recorded via 32 items scored on a 5-point Likert scale (1 = *strongly disagree*, 2 = *disagree*, 3 = *neither agree nor disagree*, 4 = *agree*, 5 = *strongly agree*). The mean and standard deviation for each of the questions are shown in Table 1. The correlation between different learning styles and perceptions of the game was also analyzed, as shown in Table 2. Out of 32 items, the majority of the perceptions ($n = 28$) did not have any significant difference, indicating the game was effective for all types of learners. In the remaining four items, significant differences were noted in Livogena: The Ikteros Curse being related to knowledge and skills ($p = .02$), having consistency in rules ($p = .04$), improving social interaction skills ($p = .02$), and being gradually challenged ($p = .01$). There was no correlation between VARK learning styles and the sex of learners.

Students' feedback was recorded on a 10-point scale (1 = *completely unhelpful*, 2 = *mostly unhelpful*, 3 = *unhelpful*, 4 = *slightly unhelpful*, 5 = *neutral*, 6 = *somewhat helpful*, 7 = *helpful*, 8 = *very helpful*, 9 = *extremely helpful*, 10 = *incredibly helpful*) as represented in the Figure. Students rated the game highly positively, with an average rating of 9.5 for development of critical thinking, future application of knowledge, sharing knowledge in groups, and self-assessment of learning gaps; 9.6 for arousing interest in active learning and recalling the concepts; and 9.7 for creating an engaging environment in which to learn. Also, students rated the game 9.6 for learning basic knowledge and differential diagnosis, 9.5 for investigations, and 9.4 for treatment for jaundice. An impressive finding is that only one response recorded any of the *unhelpful* criteria.

The individual views of students and faculty recorded for qualitative analysis also support the statistical data. Students and faculty were highly enthusiastic and motivated to learn via these kinds of activities. The responses of students and faculty were categorized into six themes: (1) ease of learning, (2) innovative approach to learning, (3) a game for assessment and feedback, (4) learning experiences, (5) role of facilitator, and (6) faculty's views. The thematic analysis of students' and faculty's responses is shown in Table 3.

Discussion

This approach of integrating stories with games was an innovative and highly effective method to facilitate the learning of jaundice. Students' ability to solve case-based questions and interpret laboratory results significantly improved after the innovation. Based on pre- and posttest results and feedback from students, it is evident that the game promotes active learning and has a positive impact on the learner. Students felt that preparing for the game was not stressful, and they enjoyed learning with it. They were also satisfied that they were able to recall, share knowledge, and identify what they had missed while learning. Thus, this innovation facilitated not only learning but also self-assessment. Students additionally perceived the gaming environment to be nonthreatening and a low-stakes evaluation. Competitiveness was also another factor motivating students to continuously engage in the game and actively learn with their peers.

In the analysis of students' perceptions and preferred learning styles, significant differences were obtained in the perception of the relatability of questions, consistency of rules, social interaction, and gradual challenges in the game. The game's connection to knowledge and skills caters to cognitive learners, consistent rules benefit those who appreciate structure, improved social interaction skills are valuable for collaborative learners, and the gradual challenge progression suits experiential and visual learners. These elements should be considered when designing future games to ensure that they are more inclusive, holistic, and accommodating to a wide range of learning preferences, thereby contributing to a richer learning experience for all players.

It is evident that gamification is a good method for provoking interest and promoting active learning among students. Games refresh the traditional approach to learning, and rekindling the fascination with fairytales and fantasy worlds among adult

Copyright © 2024 Surapaneni. This is an open-access publication distributed under the terms of the Creative Commons Attribution license.

Table 1. Perceptions of the Livogena: The Ikteros Curse Card and Board Game

Perception Survey Item	*M*	*SD*	% Strongly Agree & Agree	% Neither Agree nor Disagree	% Strongly Disagree & Disagree
The Livogena: The Ikteros Curse card and board game created a fun-filled and engaging environment to learn jaundice.	4.8	0.4	99	1	0
The questions on the Livogena: The Ikteros Curse card and board game were related to my knowledge and skills.	4.5	0.6	96	4	0
The level of the Livogena: The Ikteros Curse card and board game is tailored on the basis of the player's abilities.	4.6	0.5	98	2	0
There was consistency in the rules while playing the Livogena: The Ikteros Curse card and board game.	4.6	0.6	93	7	0
Understanding the rules and functioning of the Livogena: The Ikteros Curse card and board game was easy.	4.5	0.6	97	3	0
The complexity of the game was not harder than the learning content of the game.	4.4	0.6	93	7	0
The Livogena: The Ikteros Curse card and board game created curiosity and a sense of exploration.	4.7	0.5	97	3	0
The Livogena: The Ikteros Curse card and board game has improved my social interaction skills.	4.6	0.6	93	7	0
The Livogena: The Ikteros Curse card and board game has evoked motivation to learn jaundice.	4.6	0.5	98	2	0
The Livogena: The Ikteros Curse tool has ignited my interest towards learning jaundice in biochemistry.	4.6	0.6	97	3	0
The Livogena: The Ikteros Curse card and board game engages the player in active discovery.	4.6	0.5	100	0	0
The Livogena: The Ikteros Curse card and board game was well designed with rewards and surprises that motivated me to engage more in the game.	4.6	0.5	99	1	0
The Livogena: The Ikteros Curse card and board game is a meaningful way of learning.	4.6	0.6	96	4	0
The Livogena: The Ikteros Curse card and board game has made my learning experiential.	4.6	0.6	97	2	1
The Livogena: The Ikteros Curse card and board game is often multiplayer or social.	4.6	0.5	99	1	0
The Livogena: The Ikteros Curse card and board game can be completed within a fair time frame.	4.3	0.7	89	8	3
During the Livogena: The Ikteros Curse card and board game the players are engaged in pursuing a goal.	4.6	0.5	99	1	0
The Livogena: The Ikteros Curse card and board game is sustainable and can be easily reused at any time and place.	4.5	0.6	94	5	1
The Livogena: The Ikteros Curse card and board game has ensured immediate and effective feedback for learner development.	4.5	0.5	99	1	0
The Livogena: The Ikteros Curse card and board game fosters the ability to transfer learning from the game context to a real context.	4.6	0.5	98	2	0
The Livogena: The Ikteros Curse card and board game created a nonthreatening environment to learn.	4.6	0.5	100	0	0
The Livogena: The Ikteros Curse card and board game provides an opportunity to try, make mistakes, and learn with minimal penalty.	4.6	0.5	100	0	0
Immediate and contextualized feedback is supplied during the Livogena: The Ikteros Curse card and board game session.	4.5	0.6	96	3	1
I was able to recall what I have studied by playing the Livogena: The Ikteros Curse card and board game.	4.5	0.6	96	4	0
The Livogena: The Ikteros Curse card and board game reinforced my learning through repetition of information in lectures and textbooks.	4.6	0.5	98	2	0
During the Livogena: The Ikteros Curse card and board game the player can assess the acquired knowledge or skill with other players.	4.5	0.6	97	2	1
The Livogena: The Ikteros Curse card and board game served as a platform to test my knowledge rather than mere representation of facts.	4.6	0.5	99	1	0
The competition in the Livogena: The Ikteros Curse card and board game has encouraged me to perform better.	4.6	0.5	100	0	0
The Livogena: The Ikteros Curse card and board game has facilitated collaborative learning.	4.6	0.5	99	1	0
During the Livogena: The Ikteros Curse card and board game players are gradually challenged because they cannot move freely among the game levels.	4.4	0.6	97	2	1
Solving the Livogena: The Ikteros Curse card and board game required integration of knowledge and skills from all types of learners.	4.6	0.5	100	0	0
Completing the Livogena: The Ikteros Curse card and board game as an innovative gamification tool has created satisfaction and a sense of accomplishment in me.	4.6	0.5	100	0	0

Copyright © 2024 Surapaneni. This is an open-access publication distributed under the terms of the Creative Commons Attribution license.

Table 2. Correlation Between Perceptions and Different Types of Learning Styles

Perception Survey Item	*M* (*SD*) Visual (*N* = 11)	Aural (*N* = 7)	Read/Write (*N* = 4)	Kinesthetic (*N* = 30)	Multimodal (*N* = 51)	*p*
The Livogena: The Ikteros Curse card and board game created a fun-filled and engaging environment to learn jaundice.	4.7 (0.5)	4.7 (0.6)	4.7 (0.5)	4.6 (0.6)	4.9 (0.4)	.24
The questions on the Livogena: The Ikteros Curse card and board game were related to my knowledge and skills.	4.7 (0.5)	4.0 (0.0)	4.6 (0.5)	4.4 (0.5)	4.6 (0.7)	.02
The level of the Livogena: The Ikteros Curse card and board game is tailored on the basis of the player's abilities.	4.7 (0.5)	4.3 (0.6)	4.6 (0.5)	4.4 (0.6)	4.6 (0.6)	.49
There was consistency in the rules while playing the Livogena: The Ikteros Curse card and board game.	4.7 (0.6)	3.7 (0.6)	4.3 (0.6)	4.6 (0.6)	4.6 (0.6)	.04
Understanding the rules and functioning of the Livogena: The Ikteros Curse card and board game was easy.	4.6 (0.6)	4.3 (0.6)	4.5 (0.5)	4.5 (0.5)	4.5 (0.6)	.93
The complexity of the game was not harder than the learning content of the game.	4.6 (0.6)	4.3 (0.6)	4.3 (0.5)	4.4 (0.8)	4.5 (0.6)	.69
The Livogena: The Ikteros Curse card and board game created curiosity and a sense of exploration.	4.7 (0.6)	4.7 (0.6)	4.6 (0.5)	4.5 (0.6)	4.7 (0.5)	.86
The Livogena: The Ikteros Curse card and board game has improved my social interaction skills.	4.8 (0.4)	3.7 (0.6)	4.4 (0.7)	4.6 (0.6)	4.5 (0.7)	.02
The Livogena: The Ikteros Curse card and board game has evoked motivation to learn jaundice.	4.7 (0.5)	4.3 (0.6)	4.6 (0.5)	4.5 (0.6)	4.6 (0.5)	.62
The Livogena: The Ikteros Curse tool has ignited my interest towards learning jaundice in biochemistry.	4.6 (0.5)	4.0 (0.0)	4.6 (0.6)	4.6 (0.5)	4.6 (0.6)	.30
The Livogena: The Ikteros Curse card and board game engages the player in active discovery.	4.7 (0.5)	4.0 (0.0)	4.6 (0.5)	4.5 (0.5)	4.7 (0.5)	.14
The Livogena: The Ikteros Curse card and board game was well designed with rewards and surprises that motivated me to engage more in the game.	4.7 (0.5)	4.0 (0.0)	4.6 (0.7)	4.6 (0.5)	4.7 (0.5)	.15
The Livogena: The Ikteros Curse card and board game is a meaningful way of learning.	4.9 (0.4)	4.3 (0.6)	4.3 (0.7)	4.6 (0.6)	4.6 (0.6)	.10
The Livogena: The Ikteros Curse card and board game has made my learning experiential.	4.8 (0.4)	4.0 (0.0)	4.5 (0.5)	4.5 (0.6)	4.6 (0.7)	.05
The Livogena: The Ikteros Curse card and board game is often multiplayer or social.	4.8 (0.4)	4.3 (0.6)	4.6 (0.5)	4.6 (0.5)	4.5 (0.6)	.20
The Livogena: The Ikteros Curse card and board game can be completed within a fair time frame.	4.5(0.9)	4.3 (0.6)	4.0 (0.9)	4.3 (0.6)	4.4 (0.7)	.45
During the Livogena: The Ikteros Curse card and board game the players are engaged in pursuing a goal.	4.7 (0.5)	4.7 (0.6)	4.6 (0.5)	4.5 (0.5)	4.6 (0.5)	.78
The Livogena: The Ikteros Curse card and board game is sustainable and can be easily reused at any time and place.	4.6 (0.5)	4.0 (0.0)	4.4 (0.9)	4.5 (0.6)	4.5 (0.6)	.43
The Livogena: The Ikteros Curse card and board game has ensured immediate and effective feedback for learner development.	4.7(0.5)	4.0 (0.0)	4.5 (0.5)	4.5 (0.5)	4.5 (0.6)	.23
The Livogena: The Ikteros Curse card and board game fosters the ability to transfer learning from the game context to a real context.	4.7 (0.5)	4.0 (0.0)	4.6 (0.5)	4.5 (0.6)	4.6 (0.6)	.16
The Livogena: The Ikteros Curse card and board game created a nonthreatening environment to learn.	4.7 (0.5)	4.0 (0.0)	4.5 (0.5)	4.5 (0.5)	4.7 (0.5)	.12
The Livogena: The Ikteros Curse card and board game provides an opportunity to try, make mistakes, and learn with minimal penalty.	4.6(0.5)	4.3 (0.6)	4.4 (0.5)	4.7 (0.5)	4.6 (0.5)	.34
Immediate and contextualized feedback is supplied during the Livogena: The Ikteros Curse card and board game session.	4.4 (0.7)	4.0 (0.0)	4.5 (0.7)	4.5 (0.6)	4.5 (0.6)	.50
I was able to recall what I have studied by playing the Livogena: The Ikteros Curse card and board game.	4.6 (0.5)	4.0 (0.0)	4.5 (0.5)	4.7 (0.5)	4.4 (0.7)	.20
The Livogena: The Ikteros Curse card and board game reinforced my learning through repetition of information in lectures and textbooks.	4.7 (0.5)	4.3 (0.6)	4.6 (0.7)	4.6 (0.5)	4.6 (0.5)	.83
During the Livogena: The Ikteros Curse card and board game the player can assess the acquired knowledge or skill with other players.	4.7 (0.5)	4.0 (0.0)	4.4 (1.2)	4.5 (0.5)	4.5 (0.6)	.17
The Livogena: The Ikteros Curse card and board game served as a platform to test my knowledge rather than mere representation of facts.	4.7 (0.5)	4.0 (0.0)	4.6 (0.5)	4.5 (0.5)	4.6 (0.5)	.18
The competition in the Livogena: The Ikteros Curse card and board game has encouraged me to perform better.	4.8 (0.4)	4.3 (0.6)	4.5 (0.5)	4.5 (0.5)	4.5 (0.5)	.25
The Livogena: The Ikteros Curse card and board game has facilitated collaborative learning.	4.8 (0.5)	4.0 (0.0)	4.5 (0.5)	4.5 (0.5)	4.6 (0.5)	.06
During the Livogena: The Ikteros Curse card and board game players are gradually challenged because they cannot move freely among the game levels.	4.7 (0.5)	3.7 (0.6)	4.2 (0.4)	4.5 (0.5)	4.3 (0.6)	.01
Solving the Livogena: The Ikteros Curse card and board game required integration of knowledge and skills from all types of learners.	4.7 (0.5)	4.0 (0.0)	4.5 (0.5)	4.5 (0.5)	4.5 (0.5)	.13
Completing the Livogena: The Ikteros Curse card and board game as an innovative gamification tool has created satisfaction and a sense of accomplishment in me.	4.8 (0.4)	4.0 (0.0)	4.5 (0.5)	4.6 (0.5)	4.6 (0.5)	.09

Copyright © 2024 Surapaneni. This is an open-access publication distributed under the terms of the Creative Commons Attribution license.

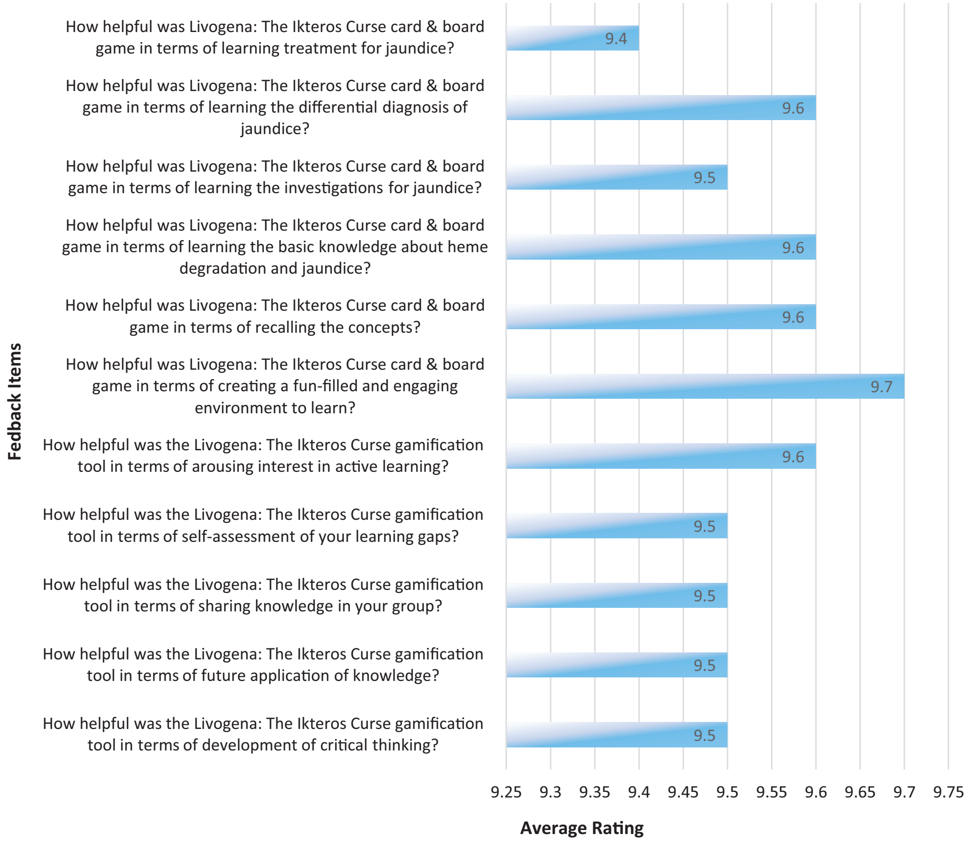

Figure. Feedback regarding the Livogena: The Ikteros Curse gamification tool. Items were rated on a 10-point scale (1 = *completely unhelpful*, 10 = *incredibly helpful*).

learners can stimulate their interest and allow them to explore the learning content with curiosity. Thus, within a stipulated time, students can learn, assess, get feedback, and also have fun while learning. While developing and implementing this innovation, the main challenge was to design a creative and relevant game model. This was the only time-consuming process. Now that it has been developed, the game is easily reproducible and does not require any additional setup apart from the cost of printing the game materials. Because this is a multiplayer, on-site game, institutions must ensure sufficient human resources. Also, while implementing this game, institutions should make sure the content is pertinent to their curriculum. The playing cards are currently up to date (at the time of publication). However, owing to rapidly growing management strategies, a few cards may become outdated. Thus, updating the content may be required in the long run. The entire time required, including faculty orientation, students' pretest and introductory video, actual gameplay, assessment, and postgame discussion, is 3 hours. However, for more efficient use of time, clear instructions and guidelines regarding the game and game materials should be provided.

The evaluation methods included perceptions and feedback assessment (Kirkpatrick level 1), improvement in learning (Kirkpatrick level 2), and assessment of behavior (Kirkpatrick level 3). The notable increase in mean scores and the highly significant statistical analysis for the intervention batch suggest that the game-based intervention had a positive and impactful effect on learners. These results demonstrate students' enhanced ability to retain and apply knowledge, showcasing the game's effectiveness in facilitating long-term comprehension and practical application of the subject matter.

Highly positive and significant responses indicate the effectiveness of such game-based innovations in medical education. Students learn effectively when they actively interact, discover, assess, and improvise on feedback given. When all such essential components can be integrated into a game, it will certainly serve as an innovative learning tool that can be incorporated into the curriculum. Games can also be implemented as assessment tools, where the purpose shifts from assessment of learning to assessment as learning and for learning. Thus, educators and policymakers should encourage

Copyright © 2024 Surapaneni. This is an open-access publication distributed under the terms of the Creative Commons Attribution license.

Table 3. Thematic Analysis of Feedback From Students and Faculty

Theme	Quotes
Ease of learning	• "The game was more fun and interesting. It helped us gain more clear understanding regarding the concepts."<br>• "Waiting for newer chapters in Livogena because studying for a game makes happy learning without stress."<br>• "It was really nice, and I not only enjoyed it but was also able to learn those chapters easily since biochemistry is a difficult subject for me."<br>• "Learning is not boring and stressful now; I would love to learn like this."
Innovative approach to learning	• "It is an interesting method and a different exposure to learning. Even in future, we need such activities to be conducted."<br>• "Please continue with these types of games for all the important topics so that we take more interest in learning which will help us score well in exams too. Livogena theme was wondrous, please come up with more different ideas like these for other topics."<br>• "This learning approach was a game-changer. I not only learned effectively but also enjoyed the process. It's left me excited for future learning experiences that incorporate innovative methods like these."
A game for assessment and feedback	• "This game was amazing. It was helpful to test my knowledge about jaundice. This game helps interactive learning. The little activity in between the game, boosts us to proceed more and more."<br>• "The game was a fantastic tool for assessing my knowledge. The immediate feedback after each challenge allowed me to identify areas where I needed improvement and adjust on the spot."<br>• "The game's assessment components were like mini checkpoints along the way. They kept me engaged and motivated to keep learning and improving my knowledge."
Learning experiences	• "It was very nice to play, and we were able to learn and enjoy at the same time."<br>• "I am able to learn without getting anxious and fear of marks."<br>• "I came to know different patterns of questions and what to concentrate on while learning."
Role of facilitator	• "The facilitator acted as a guide throughout the game, helping us navigate challenges and offering support when needed."<br>• "Our facilitator was like a guiding light during the game. They kept us on track, answered our questions, and created a supportive learning atmosphere."<br>• "Our facilitator brought a sense of structure to the game. They were there to ensure we stayed on the right path, making the learning experience more organized."
Faculty's views	• "Facilitating 'Livogena' was a unique experience. I observed how engaged and enthusiastic the students became during the game. It was clear that this approach added a layer of excitement to their learning, and I believe it deepened their understanding of jaundice."<br>• "The game offered an innovative way to teach complex medical concepts. I was pleasantly surprised to see how well the students grasped the subject matter through this interactive and narrative-driven approach. Cooperative learning is an essential component here."<br>• "I found the game to be an effective tool for student assessment. The in-game challenges and feedback mechanisms allowed us to understand their comprehension in real-time. It was refreshing to witness their active participation in assessments."<br>• "One of the standout aspects of 'Livogena' was how it fostered a collaborative learning environment. Students were actively discussing strategies, asking questions, and problem-solving together. It created a sense of camaraderie among them, which was wonderful to observe."<br>• "Facilitating the game was a fulfilling experience. It not only enriched the students' learning but also challenged me to think more creatively about my teaching methods. I believe that integrating similar approaches into medical education can be highly beneficial."

more innovations and experimentation in this rapidly evolving field.

Following the success of this innovation and students' requests to implement its approach with other topics, more games are anticipated to be designed in the future, particularly ones integrated with the component of a story and more vigorous assessment of learning objectives in addition to pre/post multiple-choice questions. Other institutions are encouraged to implement such student-centered innovations to increase learner engagement and promote active learning in medical education. However, as a limitation, it should be noted that this game was implemented in just a single institution with only one batch of students. For better generalizability, continued quality improvement through potential refinements, updating content as necessary, and ensuring the resource remains valuable for students are important. This involves regularly assessing and evaluating the impact of these innovations on student learning outcomes. It also requires collecting feedback from students, faculty, and other stakeholders and using this feedback to refine and improve the teaching methods and resources.

Appendices

A. Pre- and Posttest.doc
B. Game Instructions.doc
C. Game Story.doc
D. Game Video.mp4
E. Game Board.jpg
F. Game Cards.docx
G. Questions and Answer Key.doc
H. Game Activities.doc
I. Perceptions Questionnaire.doc
J. Feedback Questionnaire.doc

All appendices are peer reviewed as integral parts of the Original Publication.

Krishna Mohan Surapaneni, PhD, MHPE: Professor, Department of Biochemistry, and Head, Department of Medical Education, Panimalar

Copyright © 2024 Surapaneni. This is an open-access publication distributed under the terms of the Creative Commons Attribution license.

Medical College Hospital & Research Institute; ORCID: https://orcid.org/0000-0002-5204-5708

Acknowledgments

I would like to express my sincere gratitude to my Third Professional: Part 1 undergraduate medical students Ms. Arthana Jaikumar, Ms. Shivani B., Mr. Manjunath Dhanapal, Ms. Sarikasri S., Ms. Sreenidhi Prakash, and Ms. Jyotsna Needamangalam Balaji and Second Professional undergraduate medical student Mr. Abel Stanes at the Panimalar Medical College Hospital & Research Institute for their support in making the video narrative.

I thank my colleagues Dr. Anusha R., Dr. Lakshmi G., Dr. Shalini L., Ms. Saranya S., Dr. Dinesh Kumar R., and Dr. Kalaiselvi R. at the Department of Biochemistry, Panimalar Medical College Hospital & Research Institute. I thank my colleagues from the Pre-Clinical and Para-Clinical Departments, Dr. Kalyani M., Dr. Jayanthi S., Dr. Mahima Sophia M., Dr. Bhuveneswari C., Dr. Shifa Meharaj, Dr. Somasunder V.M., Dr. Mahila S., Dr. Latha, Dr. Nathini Devi P., Dr. Harshada Valvoikar, Dr. Anitha E., Dr. Neevedha Kannan, Dr. Vasanthi C., Dr. Priya G., Dr. Elavarsi P., Dr. Dinesh Kumar R., Dr. Kalaiselvi R., Dr. Preethy R., Ms. Meghna S. Manohar, Ms. Nathini, Ms. Sandhiya K., Ms. Vishvathi S., Mr. Jim Jebastin, Dr. Midhun Sakravarthy J., and Dr. Poorna Sadagopan, at the Panimalar Medical College Hospital & Research Institute for volunteering to be the faculty in charge of 25 small groups in this study. I would also like to thank Dr. Porchelvan S., Professor of Biostatistics, Department of Community Medicine, Panimalar Medical College Hospital & Research Institute, for his assistance in the statistical analysis.

Disclosures

None to report.

Funding/Support

None to report.

Ethical Approval

The Panimalar Medical College Hospital & Research Institute Institutional Human Ethics Committee approved this project.

References

1. Abbas MW, Shamshad T, Ashraf MA, Javaid R. Jaundice: a basic review. *Int J Res Med Sci*. 2016;4(5):1313-1319. https://doi.org/10.18203/2320-6012.ijrms20161196
2. Xu M, Luo Y, Zhang Y, Xia R, Qian H, Zou X. Game-based learning in medical education. *Front Public Health*. 2023;11: 1113682. https://doi.org/10.3389/fpubh.2023.1113682
3. Gorbanev I, Agudelo-Londoño S, González RA, et al. A systematic review of serious games in medical education: quality of evidence and pedagogical strategy. *Med Educ Online*. 2018;23(1):1438718. https://doi.org/10.1080/10872981.2018.1438718
4. Krishnamurthy K, Selvaraj N, Gupta P, et al. Benefits of gamification in medical education. *Clin Anat*. 2022;35(6): 795-807. https://doi.org/10.1002/ca.23916
5. Walker J, Heudebert JP, Patel M, et al. Leveraging technology and gamification to engage learners in a microbiology curriculum in undergraduate medical education. *Med Sci Educ*. 2022;32(3):649-655. https://doi.org/10.1007/s40670-022-01552-7
6. Sannathimmappa MB, Nambiar V, Aravindakshan R. Learning out of the box: fostering intellectual curiosity and learning skills among the medical students through gamification. *J Educ Health Promot*. 2022;11(1):79.
7. Gue S, Ray J, Ganti L. Gamification of graduate medical education in an emergency medicine residency program. *Int J Emerg Med*. 2022;15:41. https://doi.org/10.1186/s12245-022-00445-1
8. Yashaswini S, Shylaja SS. Story telling: learning to visualize sentences through generated scenes. In: Shukla PK, Singh KP, Tripathi AK, Engelbrecht A, eds. *Computer Vision and Robotics: Algorithms for Intelligent Systems*. Springer; 2023:1-14. https://doi.org/10.1007/978-981-19-7892-0_1
9. Watsjold B, Zhong D. Clinical Coaching Cards: a game of active learning theory and teaching techniques. *MedEdPORTAL*. 2020;16:11042. https://doi.org/10.15766/mep_2374-8265.11042
10. Cusick J. A Jeopardy-style review game using team clickers. *MedEdPORTAL*. 2016;12:10485. https://doi.org/10.15766/mep_2374-8265.10485
11. Ellis G. Pharmacotherapy update and review for family medicine residents using Jeopardy-style game. *MedEdPORTAL*. 2020;16: 10941. https://doi.org/10.15766/mep_2374-8265.10941
12. Bridges EP, Foster CE, Park DB, Lehman-Huskamp KL, Mark DW, Tuuri RE. Learning to beat the shock clock: a low-fidelity simulation board game for pediatric and emergency medicine residents. *MedEdPORTAL*. 2019;15:10804. https://doi.org/10.15766/mep_2374-8265.10804
13. Pisano TJ, Santibanez V, Hernandez M, Patel D, Osorio G. The bloody board game: a game-based approach for learning high-value care principles in the setting of anemia diagnosis. *MedEdPORTAL*. 2020;16:11057. https://doi.org/10.15766/mep_2374-8265.11057
14. Hill RV, Nassrallah Z. A game-based approach to teaching and learning anatomy of the liver and portal venous system. *MedEdPORTAL*. 2018;14:10696. https://doi.org/10.15766/mep_2374-8265.10696
15. Woosley J, Premkumar K, Staples E. Medical solitaire: a flash-based game facilitating study and review of lecture content. “Cysts and tumors of the liver.” *MedEdPORTAL*. 2010;6:7899. https://doi.org/10.15766/mep_2374-8265.7899

Copyright © 2024 Surapaneni. This is an open-access publication distributed under the terms of the Creative Commons Attribution license.

16. Medical Council of India. *Competency Based Undergraduate Curriculum for the Indian Medical Graduate*. Vol 1. Medical Council of India; 2018. Accessed December 21, 2023. https://www.nmc.org.in/wp-content/uploads/2020/01/UG-Curriculum-Vol-I.pdf

17. Fleming ND, Mills C. Not another inventory, rather a catalyst for reflection. *To Improv Acad*. 1992;11(1):137-155. https://doi.org/10.1002/j.2334-4822.1992.tb00213.x

18. The VARK Questionnaire—how do you learn best? *VARK*. Accessed December 21, 2023. https://vark-learn.com/the-vark-questionnaire/

19. Kirkpatrick JD, Kirkpatrick WK. *Kirkpatrick's Four Levels of Training Evaluation*. ATD Press; 2016.

Received: July 20, 2023
Accepted: November 14, 2023
Published: February 6, 2024

Copyright © 2024 Surapaneni. This is an open-access publication distributed under the terms of the Creative Commons Attribution license.
