# Supplementary figures and images for: Livogena: The Ikteros Curse—A Jaundice Narrative Card and Board Game for Medical Students
Source: MedEdPORTAL. 2024 Feb 6;20:11381. doi: 10.15766/mep_2374-8265.11381 (PMC10844581; doi:10.15766/mep_2374-8265.11381)

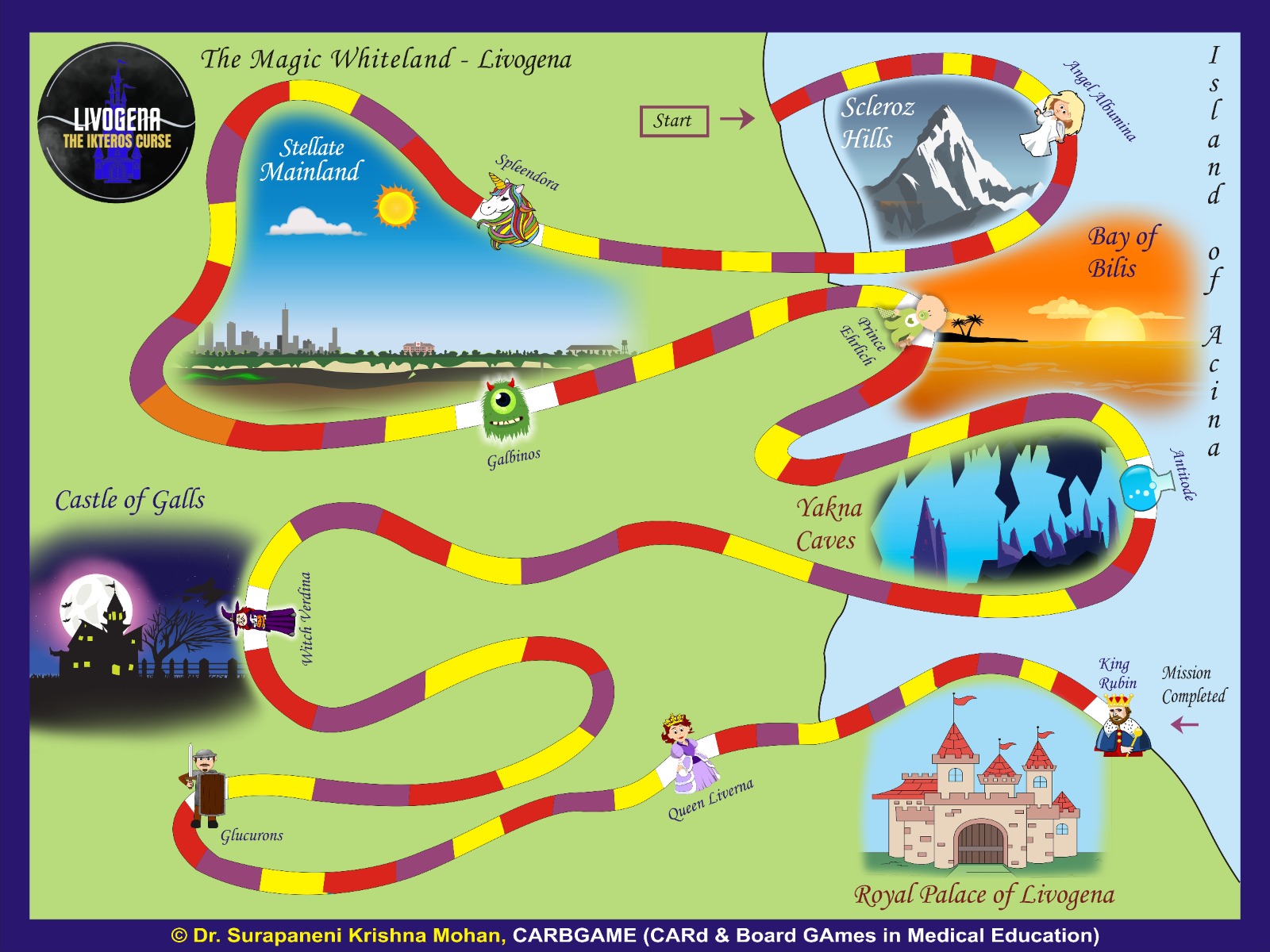

Supplement: Supplementary file 1 — Pre- and Posttest.docGame Instructions.docGame Story.docGame Video.mp4Game Board.jpgGame Cards.docxQuestions and Answer Key.docGame Activities.docPerceptions Questionnaire.docFeedback Questionnaire.doc [file mep_2374-8265.11381-s001.zip › E. Game Board.jpg]
