# Supplementary material for: Livogena: The Ikteros Curse—A Jaundice Narrative Card and Board Game for Medical Students
Source: MedEdPORTAL. 2024 Feb 6;20:11381. doi: 10.15766/mep_2374-8265.11381 (PMC10844581; doi:10.15766/mep_2374-8265.11381)
Supplement: Supplementary file 1 — Pre- and Posttest.docGame Instructions.docGame Story.docGame Video.mp4Game Board.jpgGame Cards.docxQuestions and Answer Key.docGame Activities.docPerceptions Questionnaire.docFeedback Questionnaire.doc [file mep_2374-8265.11381-s001.zip › H. Game Activities.doc]

# LIVOGENA: “THE IKTEROS CURSE”

# Note: These are the activities for each character. This should be displayed to students, preferably in printed form for them to complete. The answer to each activity is provided at the end. The facilitator can have a copy of the answers separately to cross-check. The time required for each activity is 5 minutes.

# ACTIVITIES FOR CHARACTERS

**ACTIVITY 1**

**Goal:** To perform the activity and please Angel Albumina so that she will travel along with you to guide you in this rescue mission

**Activity: ACT & ATTRACT**

Attract Angel Albumina with your acting skills. It's DUMB CHARADES. You will be given 3 words. The faculty in charge will disclose the words to the player who is going to enact them. One player from the team enacts and the others should guess the answer. No verbal signs are allowed. The same player can act thrice, or players can take turns to act. Once you find all three diseases, you have the angel by your side!!

# ACTIVITY 2

**Goal:** To complete the task and gain the trust of Spleendora to take you to the Yakna caves by helping you cross the Bay of Bilis

**Activity: CAPTURE HER ATTENTION**

Spleendora is an extremely beautiful unicorn who is proud of its beauty. She doesn’t come in front of unknown people. But she loves people who pay attention to small details. It's now your turn to capture her attention by completing this task so that she will trust your skills and help you cross the Bay of Bilis

A sheet with 25 images will be kept in front of you. Any two players shall play first. You have 10 seconds to observe the picture. Once the time is up, you will have to tell the names of the images that you have seen within 30 seconds. You need to tell at least 20 correct names within 30 seconds to complete this task. The faculty in charge shall check the list as the players tell the names. If one sub-team is unable to complete, the next two players shall play again, and this will continue until you complete the task. Note that when two players are observing the sheet, the other players shall not look into it. The timings will be monitored by the faculty in charge.

| Figure 1   | Figure 2   | Figure 3   | Figure 4   | Figure 5   |
| --- | --- | --- | --- | --- |
| Figure 6   | Figure 7   | Figure 8  INCLUDEPICTURE "../Library/Group%20Containers/UBF8T346G9.ms/WebArchiveCopyPasteTempFiles/com.microsoft.Word/nail-clipper-clipart-md.png" \* MERGEFORMAT  | Figure 9   | Figure 10  INCLUDEPICTURE "../Library/Group%20Containers/UBF8T346G9.ms/WebArchiveCopyPasteTempFiles/com.microsoft.Word/calender-2389150_1280.png" \* MERGEFORMAT  |
| Figure 11   | Figure 12   | Figure 13   | Figure 14   | Figure 15  INCLUDEPICTURE "../Library/Group%20Containers/UBF8T346G9.ms/WebArchiveCopyPasteTempFiles/com.microsoft.Word/band-aid-3116999_1280.png" \* MERGEFORMAT  |
| Figure 16   | Figure 17   | Figure 18  INCLUDEPICTURE "../Library/Group%20Containers/UBF8T346G9.ms/WebArchiveCopyPasteTempFiles/com.microsoft.Word/bunsen-burner-154235_1280.png" \* MERGEFORMAT  | Figure 19   | Figure 20   |
| Figure 21   | Figure 22   | Figure 23   | Figure 24   | Figure 25   |

# ACTIVITY 3

**Goal:** To defeat the evil monsters Galbinos who are causing havoc in the Stellate Mainland and cross the city.

**Activity: CONNECT & CONFUSE**

The aim of this activity is to confuse the Galbinos by connecting all the things that are yellow in color. As the Galbinos get confused, they will start losing their power and you can easily escape out of the Stellate Mainland to reach the Bay of Bilis.

All the players of the team shall randomly start telling the name of anything that is yellow in color one by one. The players shall go in the same order and tell a thing that is yellow in color within 5 seconds. Players who run out of words will be eliminated one by one. The game shall continue until the two players compete in the last round. The winner of this final round will be the one who defeated the Galbinos.

The faculty in charge shall monitor the game and timings and make sure that the words are not repeated.

# ACTIVITY 4

**Goal:** To cross the Bay of Bilis on winged Spleendora and rescue newborn prince Ehrlich who is hidden inside the bay.

**Activity: LETTERS IN A STRING**

You are now in the middle of Bay of Bilis. Angel Albumina has found that prince Ehrlich is hidden inside this bay. In order to rescue prince Ehrlich, you will have to find the three code words which will be split and depicted as images. Find the image and guess the word to rescue Prince Ehrlich

1.

| Figure 26   | Figure 27   | Figure 28   |
| --- | --- | --- |

**2.**

| Figure 29   | Figure 30   | Figure 31   |
| --- | --- | --- |

3.

| Figure 32   | Figure 33   | Figure 34   | Figure 35   |
| --- | --- | --- | --- |

# ACTIVITY 5

**Goal:** To solve the puzzle and unlock the cave which hides the antidote that you are seeking

**Activity: DESTINY REWRITTEN**

Its time you find the antidote to the Ikteros poison. You have reached the Yakna Caves but the Cave unlocks only upon finding the answer to the riddle. Take this advice and solve the riddle to get the antidote that you seek.

*“It may look easy to comprehend*

*But it is always not the end*

*What you find may be right*

*But it may also put you in a plight*

*Search your mind for you know*

*And the way to antidote soon will show”*

RIDDLE:

**I'm not a mineral, nor a gem,**

**But I'm vital to life, like a precious stem.**

**I help clean up the blood and break down fat,**

**Without me, your body would be quite flat.**

# WHAT IS THE PIN?

**ACTIVITY 6**

**Goal:** To defeat witch Verdina in her castle so that the iketros curse on the kingdom will be abolished

**Activity: HANG THE WITCH**

Now it’s the most crucial time. You need to defeat the witch. The game is similar to HANGMAN with little changes. Here, for every correct letter that you find, the head and other lines will be drawn one by one such that a complete figure given below is drawn. Once you find the 11-lettered word, the witch will be hanged! In the figure, there are 9 lines and 1 head corresponding to the other 10 letters that you have to find

[Figure 36]

N

# ACTIVITY 7

**Goal:** To complete the activity and obtain the permission of the royal guards Glucurons and enter the royal palace.

**Activity: CRYSTALS CODE**

You’re almost there. As you have defeated the witch, the spell on the royal guards has vanished. They protect the royal palace from enemies. Prove that you are here to help. For the following statements, state true or false. True corresponds to number 1 and false corresponds to 0. Only after getting the correct 5-digit crystal code, you will be allowed to enter the palace.

1. Free Bilirubin is always absent in urine
2. Conjugated bilirubin is insoluble in water
3. Free bilirubin is present in the bile
4. Conjugated bilirubin is not absorbed from the gastrointestinal tract
5. Free bilirubin diffuses into tissues

# ENTER THE CRYSTAL CODE

**ACTIVITY 8**

**Goal:** To enter the royal chamber of Queen Liverna and save her by giving the antidote to the poison

**Activity: CURE THE QUEEN**

Bravo! You have made your entry into the royal palace. The queen is severely ill. It is now your task to find the type of Ikeros poisoning (type of jaundice) in the queen so that you can adjust the dose of the Hepasoteria drug for quick recovery. Here is the case history of our beloved Queen, Liverna

Queen Liverna has consumed the Ikteros poison which has led to nausea, vomiting and fatigue. Her blood and urinary findings are below

**Serum bilirubin—12.0 mg%**

**Conjugated bilirubin—2 mg%**

**Unconjugated bilirubin—10 mg%**

**SGOT—20 U/L**

# SGPT—32 U/L

# ALP— 45 U/L

# LDH – 500 U/L

**Urine:**

**Bile salts—Negative,**

**Bile pigments—Negative, Urobilinogen—Positive (+++)**

**Feces stercobilinogen—Positive (+++) What is the type of Ikteros poisoning?**

# ACTIVITY 9

**Goal:** To save King Rubin from the Ikteros poison by giving the antidote and complete the game

**Activity: SAVE THE KING**

You are almost nearing to win this game! You have saved the queen. It is now time for you to save the king. In the same way how, you have saved the queen, find the type of Ikteros poisoning in the king and save him from this curse. Here is the case of our Great King, Rubin

King Rubin has been poisoned with the Ikteros potion and suffering from abdominal pain, fatigue, yellow discoloration of sclera and complains of passing pale stools. He was recently treated for cholecystitis (inflammation of the gallbladder). His blood and urinary findings are below

**Serum bilirubin—10.0 mg%**

**Conjugated bilirubin—9.4 mg%**

**Unconjugated bilirubin— 0.6 mg%**

**SGOT—75 U/L**

# SGPT—95 U/L

# ALP—490 U/L

**Urine:**

**Bile salts—Positive (++),**

**Bile pigments—Positive (++), Urobilinogen—Negative**

**Feces stercobilinogen—Negative What is the type of Ikteros poisoning?**

# ANSWERS FOR ACTIVITIES

**ACTIVITY 1:**

**Words:**

1. Carcinoma of head of pancreas
2. Thalassemia
3. Road traffic accident

# ACTIVITY 2:

**List of images**

1. Liver

2. Capsule

3. Syringe

4. Vial

5. RBC

6. Gloves

7. Mask

8. Nail cutter

9. Surgical scissors

10. Calendar

11. Spleen

12. Surgical knife

13. Blood bag

14. Test tubes

15. Band-aid

16. Lemon

17. Dissection forceps

18. Bunsen burner

19. Conical filter

20. The human heart

21. Biohazard Symbol

22. Ambulance

23. Glucose

24. Thermometer

25. Electrocardiogram

# ACTIVITY 3:

All things that are yellow in color Make sure there is no repetition Only 5 seconds allowed to think

# ACTIVITY 4:

**Answers:**

1. Bilirubin – BILL- RUBY-INN
2. Malaria – MALL-AIR-RHEA
3. Barbiturates- BAR-BEE-TWO-RATES

# ACTIVITY 5

**Answer:**

LIVER – PIN is corresponding numbered alphabets 12 9 22 5 18

# ACTIVITY 6

**Answer:**

CHOLANGITIS

# ACTIVITY 7

**Answer**: T F F T T Crsytal Code: 10011

# ACTIVITY 8

Pre hepatic or hemolytic type of Ikteros poisoning (jaundice)

# ACTIVITY 9

Post-hepatic or obstructive type of Ikteros poisoning (jaundice)
