# Supplementary material for: Livogena: The Ikteros Curse—A Jaundice Narrative Card and Board Game for Medical Students
Source: MedEdPORTAL. 2024 Feb 6;20:11381. doi: 10.15766/mep_2374-8265.11381 (PMC10844581; doi:10.15766/mep_2374-8265.11381)
Supplement: Supplementary file 1 — Pre- and Posttest.docGame Instructions.docGame Story.docGame Video.mp4Game Board.jpgGame Cards.docxQuestions and Answer Key.docGame Activities.docPerceptions Questionnaire.docFeedback Questionnaire.doc [file mep_2374-8265.11381-s001.zip › I. Perceptions Questionnaire.doc]

Note: This is for game evaluation. These questions should be made into an online survey and shared with students after the game.

**LEARNER'S PERCEPTIONS ON “LIVOGENA: THE IKTEROS CURSE", A CARD & BOARD GAME AS AN INNOVATIVE GAMIFICATION TOOL TO LEARN JAUNDICE IN BIOCHEMISTRY OF FIRST PROFESSIONAL MEDICAL CURRICULUM.**

1. "Livogena: The Ikteros Curse" card & board game created a fun-filled and engaging environment to learn Jaundice.

Strongly agree

Agree

Neither agree nor disagree

Disagree

Strongly disagree

2. The questions on "Livogena: The Ikteros Curse" card & board game was related to my knowledge and skills

Strongly agree

Agree

Neither agree nor disagree

Disagree

Strongly disagree

3. The level of the "Livogena: The Ikteros Curse" card & board game is tailored on the basis of the player's abilities.

Strongly agree

Agree

Neither agree nor disagree

Disagree

Strongly disagree

4. There was consistency in the rules while playing the "Livogena: The Ikteros Curse" card & board game.

Strongly agree

Agree

Neither agree nor disagree

Disagree

Strongly disagree

5. Understanding the rules and functioning of the "Livogena: The Ikteros Curse" card & board game was easy.

Strongly agree

Agree

Neither agree nor disagree

Disagree

Strongly disagree

6. The complexity of the game was not harder than the learning content of the game.

Strongly agree

Agree

Neither agree nor disagree

Disagree

Strongly disagree

7. The "Livogena: The Ikteros Curse" card & board game created curiosity and sense of exploration.

Strongly agree

Agree

Neither agree nor disagree

Disagree

Strongly disagree

8. The "Livogena: The Ikteros Curse" card & board game has improved my social interaction skills.

Strongly agree

Agree

Neither agree nor disagree

Disagree

Strongly disagree

9. The "Livogena: The Ikteros Curse" card & board game has evoked motivation to learn Jaundice.

Strongly agree

Agree

Neither agree nor disagree

Disagree

Strongly disagree

10. The "Livogena: The Ikteros Curse" tool has ignited my interest towards learning Jaundice in biochemistry.

Strongly agree

Agree

Neither agree nor disagree

Disagree

Strongly disagree

11. The "Livogena: The Ikteros Curse" card & board game engages the player in active discovery.

Strongly agree

Agree

Neither agree nor disagree

Disagree

Strongly disagree

12. The "Livogena: The Ikteros Curse" card & board game was well-designed with rewards and surprises that motivated me to engage more in the game.

Strongly agree

Agree

Neither agree nor disagree

Disagree

Strongly disagree

13. The "Livogena: The Ikteros Curse" card & board game is a meaningful way of learning.

Strongly agree

Agree

Neither agree nor disagree

Disagree

Strongly disagree

14. The "Livogena: The Ikteros Curse" card & board game has made my learning experiential.

Strongly agree

Agree

Neither agree nor disagree

Disagree

Strongly disagree

15. The "Livogena: The Ikteros Curse" card & board game is often multiplayer or social.

Strongly agree

Agree

Neither agree nor disagree

Disagree

Strongly disagree

16. The "Livogena: The Ikteros Curse" card & board board game can be completed within a fair time frame.

Strongly agree

Agree

Neither agree nor disagree

Disagree

Strongly disagree

17. During the "Livogena: The Ikteros Curse" card & board game the players are engaged in pursuing a goal.

Strongly agree

Agree

Neither agree nor disagree

Disagree

Strongly disagree

18. The "Livogena: The Ikteros Curse" card & board game is sustainable and can be easily reused at any time and place.

Strongly agree

Agree

Neither agree nor disagree

Disagree

Strongly disagree

19. The "Livogena: The Ikteros Curse" card & board game has ensured immediate and effective feedback for learner development.

Strongly agree

Agree

Neither agree nor disagree

Disagree

Strongly disagree

20. The "Livogena: The Ikteros Curse" card & board game fosters the ability to transfer learning from the game context to a real context.

Strongly agree

Agree

Neither agree nor disagree

Disagree

Strongly disagree

21. The "Livogena: The Ikteros Curse" card & board game created a non-threatening environment to learn.

Strongly agree

Agree

Neither agree nor disagree

Disagree

Strongly disagree

22. The "Livogena: The Ikteros Curse" card & board game provides an opportunity to try, make mistakes and learn with minimal penalty.

Strongly agree

Agree

Neither agree nor disagree

Disagree

Strongly disagree

23. Immediate and contextualized feedback is supplied during the "Livogena: The Ikteros Curse" card & board game session.

Strongly agree

Agree

Neither agree nor disagree

Disagree

Strongly disagree

24. I was able to recall what I have studied by playing the "Livogena: The Ikteros Curse" card & board game.

Strongly agree

Agree

Neither agree nor disagree

Disagree

Strongly disagree

25. The "Livogena: The Ikteros Curse" card & board game reinforced my learning through repetition of information in lectures and textbook.

Strongly agree

Agree

Neither agree nor disagree

Disagree

Strongly disagree

26. During the "Livogena: The Ikteros Curse" card & board game the player can assess the acquired knowledge or skill with other players.

Strongly agree

Agree

Neither agree nor disagree

Disagree

Strongly disagree

27. The "Livogena: The Ikteros Curse" card & board game served as a platform to test my knowledge rather than mere representation of facts.

Strongly agree

Agree

Neither agree nor disagree

Disagree

Strongly disagree

28. The competition in the "Livogena: The Ikteros Curse" card & board game has encouraged me to perform better.

Strongly agree

Agree

Neither agree nor disagree

Disagree

Strongly disagree

29. The "Livogena: The Ikteros Curse" card & board game has facilitated collaborative learning.

Strongly agree

Agree

Neither agree nor disagree

Disagree

Strongly disagree

30. During the "Livogena: The Ikteros Curse" card & board game players are gradually challenged because they can not move freely among the game levels.

Strongly agree

Agree

Neither agree nor disagree

Disagree

Strongly disagree

31. Solving the "Livogena: The Ikteros Curse" card & board game required integration of knowledge and skills from all types of learners.

Strongly agree

Agree

Neither agree nor disagree

Disagree

Strongly disagree

32. Completing the "Livogena: The Ikteros Curse" card & board game as an innovative gamification tool has created satisfaction and sense of accomplishment in me.

Strongly agree

Agree

Neither agree nor disagree

Disagree

Strongly disagree

33. Do you wish to make any additional comments?
