# Supplementary material for: Livogena: The Ikteros Curse—A Jaundice Narrative Card and Board Game for Medical Students
Source: MedEdPORTAL. 2024 Feb 6;20:11381. doi: 10.15766/mep_2374-8265.11381 (PMC10844581; doi:10.15766/mep_2374-8265.11381)
Supplement: Supplementary file 1 — Pre- and Posttest.docGame Instructions.docGame Story.docGame Video.mp4Game Board.jpgGame Cards.docxQuestions and Answer Key.docGame Activities.docPerceptions Questionnaire.docFeedback Questionnaire.doc [file mep_2374-8265.11381-s001.zip › J. Feedback Questionnaire.doc]

Note: This is for game evaluation. These questions should be made into an online survey and shared with students after the game.

**LEARNER'S FEEDBACK ON “LIVOGENA: THE IKTEROS CURSE", A CARD & BOARD GAME AS AN INNOVATIVE GAMIFICATION TOOL TO LEARN JAUNDICE IN BIOCHEMISTRY OF FIRST PROFESSIONAL MEDICAL CURRICULUM**

1 – Completely unhelpful

2 - Mostly Unhelpful

3 - Unhelpful

4 - Slightly Unhelpful

5 - Neutral

6 - Somewhat Helpful

7 - Helpful

8 - Very Helpful

9 - Extremely Helpful

10 - Incredibly Helpful

1. How helpful was the “Livogena-The Ikteros Curse” gamification tool in terms of development of critical thinking?

| 1 | 2 | 3 | 4 | 5 | 6 | 7 | 8 | 9 | 10 |
| --- | --- | --- | --- | --- | --- | --- | --- | --- | --- |

2. How helpful was the “Livogena-The Ikteros Curse” gamification tool in terms of future application of knowledge?

| 1 | 2 | 3 | 4 | 5 | 6 | 7 | 8 | 9 | 10 |
| --- | --- | --- | --- | --- | --- | --- | --- | --- | --- |

3. How helpful was the “Livogena-The Ikteros Curse” gamification tool in terms of sharing knowledge in your group?

| 1 | 2 | 3 | 4 | 5 | 6 | 7 | 8 | 9 | 10 |
| --- | --- | --- | --- | --- | --- | --- | --- | --- | --- |

4. How helpful was the “Livogena-The Ikteros Curse” gamification tool in terms of self- assessment of your learning gaps?

| 1 | 2 | 3 | 4 | 5 | 6 | 7 | 8 | 9 | 10 |
| --- | --- | --- | --- | --- | --- | --- | --- | --- | --- |

5. How helpful was the “Livogena-The Ikteros Curse” gamification tool in terms of arousing interest in active learning?

| 1 | 2 | 3 | 4 | 5 | 6 | 7 | 8 | 9 | 10 |
| --- | --- | --- | --- | --- | --- | --- | --- | --- | --- |

6. How helpful was “Livogena-The Ikteros Curse” card & board game in terms of creating a fun-filled and engaging environment to learn?

| 1 | 2 | 3 | 4 | 5 | 6 | 7 | 8 | 9 | 10 |
| --- | --- | --- | --- | --- | --- | --- | --- | --- | --- |

7. How helpful was “Livogena-The Ikteros Curse” card & board game in terms of recalling the concepts?

| 1 | 2 | 3 | 4 | 5 | 6 | 7 | 8 | 9 | 10 |
| --- | --- | --- | --- | --- | --- | --- | --- | --- | --- |

8. How helpful was “Livogena-The Ikteros Curse” card & board game in terms of learning the basic knowledge about heme degradation & jaundice?

| 1 | 2 | 3 | 4 | 5 | 6 | 7 | 8 | 9 | 10 |
| --- | --- | --- | --- | --- | --- | --- | --- | --- | --- |

9. How helpful was “Livogena-The Ikteros Curse” card & board game in terms of learning the investigations for jaundice?

| 1 | 2 | 3 | 4 | 5 | 6 | 7 | 8 | 9 | 10 |
| --- | --- | --- | --- | --- | --- | --- | --- | --- | --- |

10. How helpful was “Livogena-The Ikteros Curse” card & board game in terms of learning the differential diagnosis of jaundice?

| 1 | 2 | 3 | 4 | 5 | 6 | 7 | 8 | 9 | 10 |
| --- | --- | --- | --- | --- | --- | --- | --- | --- | --- |

11. How helpful was “Livogena-The Ikteros Curse” card & board game in terms of learning treatment for jaundice?

| 1 | 2 | 3 | 4 | 5 | 6 | 7 | 8 | 9 | 10 |
| --- | --- | --- | --- | --- | --- | --- | --- | --- | --- |
