# Supplementary material for: Livogena: The Ikteros Curse—A Jaundice Narrative Card and Board Game for Medical Students
Source: MedEdPORTAL. 2024 Feb 6;20:11381. doi: 10.15766/mep_2374-8265.11381 (PMC10844581; doi:10.15766/mep_2374-8265.11381)
Supplement: Supplementary file 1 — Pre- and Posttest.docGame Instructions.docGame Story.docGame Video.mp4Game Board.jpgGame Cards.docxQuestions and Answer Key.docGame Activities.docPerceptions Questionnaire.docFeedback Questionnaire.doc [file mep_2374-8265.11381-s001.zip › A. Pre- and Posttest.doc]

LIVOGENA: “THE IKTEROS CURSE”

PRE & POST -TEST

Note: This is the material used for pre and post-test. It should be distributed to students before and after the game. The time for completion is 20 minutes.

1. **Bilirubin in serum can be measured by which of the following methods?**
   a. Van den Bergh reaction
   b. Ehrlich’s reaction
   c. Schlesinger’s reaction
   d. Fouchet’s reaction
2. **Bilirubin is formed in all tissues, except:**
   a. Liver
   b. Spleen
   c. Kidney
   d. Bone marrow
3. **The normal color of feces is due to:**
   a. Bilirubin
   b. Stercobilin
   c. Porphobilinogen
   d. Biliverdin
4. **Congenital hyperbilirubinemia with a bilirubin level of 2.5 mg/dL is suggestive of**
   a. Crigler-Najjar syndrome
   b. Gilbert syndrome
   c. Dubin-Johnson syndrome
   d. Rotor syndrome
5. **In obstructive jaundice, prothrombin time:**
   a. Remains same
   b. Decreases
   c. Becomes normal when vitamin K is administered
   d. Increases when vitamin K is administered
6. **Severity of jaundice is high in**
a. Dubin-Johnson syndrome
b. Crigler Najjar syndrome - 1
c. Gilbert syndrome
d. Crigler Najjar syndrome - 2
7. **What is the normal level of total bilirubin level in serum?**
   a. 0.3 -3 mg/dL
   b. 1.5 – 3 mg/dL
   c. 0.2-1 mg/dL

d. 0.2 – 2.5mg/dL

8. **Bilirubin formed in peripheral tissues is transported to the liver by which of the following?**
   a. Plasma globulin
   b. Plasma lipoprotein
   c. Transferrin
   d. Plasma albumin
9. **Which of the following is the rate-limiting step in the catabolism of heme**
   a. Generation of bilirubin
   b. Transport to liver
   c. Conjugation in liver
   d. Excretion of bilirubin to bile
10. **In which type of jaundice, serum alkaline phosphatase is highly elevated?**
    a. Hemolytic jaundice
    b. Obstructive jaundice
    c. Hepatocellular jaundice
    d. Gilbert syndrome
11. **A 10-year-old male presents with increased serum bilirubin (direct) and increased bilirubin in urine with no urobilinogen. The probable diagnosis is?**
    a. Hemolytic jaundice
    b. Hepatocellular jaundice
    c. Obstructive jaundice
    d. G-6-P-D deficiency
12. **A 35-year-old male presents to the OPD with dull right abdominal pain, fever, and loss of appetite. Blood and urinary findings are as below**

Serum bilirubin—13.0 mg% Conjugated bilirubin—6.0 mg% Unconjugated bilirubin—7.0 mg% Serum alkaline phosphatase—280 IU/L SGOT—250 U/L

SGPT—370 U/L

Urine:

Bile salts—Negative,

Bile pigments—Positive (+++), Urobilinogen—Positive

Feces stercobilinogen—Positive What is your probable diagnosis?

a. Hemolytic jaundice
b. Carcinoma of the head of the pancreas
c. Viral Hepatitis
d. Primary hepatoma

13. **Comment on the following laboratory results obtained in a female patient who complained of passing clay-colored stools and yellow discoloration of sclera. Give your probable diagnosis.**

Serum bilirubin—9.0 mg% Conjugated bilirubin—7.5 mg% Unconjugated bilirubin—1.5 mg% SGOT—80 U/L

SGPT—90 U/L ALP—190 KA Units

Urine:

Bile salts—Positive (++),

Bile pigments—Positive (++), Urobilinogen—Negative

Feces stercobilinogen—Negative

a. Obstructive jaundice
b. Viral Hepatitis
c. Hemolytic jaundice due to Sickle cell anemia
d. Hemolytic jaundice due to thalassemia

14. **A 40-year-old male presents with yellowish discoloration of eyes, pallor, fatigue, and loss of appetite. Laboratory findings revealed increased reticulocyte, LDH – 5000 units/ml, unconjugated bilirubin- 5mg/dL, conjugated bilirubin is 2 mg/dL and urinary urobilinogen increased. Which of the following may not be the cause of this condition?**
    a. Sickle cell anemia
    b. G-6-P-D deficiency
    c. Hereditary spherocytosis
    d. Viral Hepatitis
15. **A 21-year-old female presents to the OPD with fatigue and breathlessness. There is yellow discoloration of the skin and sclera. What would be your probable diagnosis with the given laboratory findings?**

Serum bilirubin—9.0 mg% Conjugated bilirubin—0.5 mg% Unconjugated bilirubin—8.5 mg% SGOT—26 U/L

SGPT—30 U/L

ALP—8 KA Units Urine:

Bile salts—Negative,

Bile pigments—Negative, Urobilinogen—Positive (+++)

Feces stercobilinogen—Positive (+++)

a. Hepatocellular jaundice
b. Hemolytic jaundice
c. Jaundice due to Gall stones
d. Biliary cirrhosis

16. **An 8-year-old female who was treated with sulfonamide repeatedly for urinary tract infections presented with pallor, irritability, and fatigue. O/E there was yellow discoloration of sclera. Blood investigations revealed that she is severely anemic and has elevated levels of indirect bilirubin. This drug-induced condition may be due to the deficiency of which enzyme?**
    a. Glucuronyl transferase
    b. Biliverdin reductase
    c. Glucose 6 Phosphate Dehydrogenase
    d. Heme oxygenase
17. **A newborn baby develops jaundice immediately after birth. The mother is found to be Rh- negative. The laboratory results of the newborn are as follows**

Sex of the baby: Male

Heart rate: 90 beats/ min Temperature: febrile

Icterus: positive Lymphadenopathy: Negative Total bilirubin: 8 mg/dL Indirect bilirubin: 6.5 mg/dL Direct bilirubin: 1.5 mg/dL

What will be the urinary findings of the newborn in accordance with your probable diagnosis?

a. Urine bilirubin (direct)- absent, Urobilinogen- present
b. Urine bilirubin (direct)- present, Urobilinogen- absent
c. Urine bilirubin (direct)- present, Urobilinogen- present
d. Urine bilirubin (direct)- absent, Urobilinogen- absent

18. **A 48-year-old male presents with C/O sudden intensifying pain in the upper right abdomen that radiates to the right shoulders and symptoms of jaundice. He also complained of passing clay-colored stools. His weight is 88 kilograms, and his height is 155 cm. His BMI is 37. O/E there is yellow discoloration of the sclera and skin. You assume the condition to be obstructive jaundice. What would be the expected urinary findings in this patient?**
    a. Urine bilirubin (direct)- present, Urobilinogen- present
    b. Urine bilirubin (direct)- absent, Urobilinogen- present
    c. Urine bilirubin (direct)- absent, Urobilinogen- absent
    d. Urine bilirubin (direct)- present, Urobilinogen- absent
19. **A 56-year-old female presents with symptoms of obstructive jaundice. What will be the Van den Berg test results for this patient?**
    a. Indirect positive
    b. Direct positive
    c. Biphasic reaction
    d. No change
20. **In all newborns, mild jaundice appears after the 2nd day of life. Thus, serum bilirubin level is checked in all newborns on the 3rd day after birth. Usually, it lies in the range of 8- 12 mg/dL What would be the most appropriate management if there is a rise in serum bilirubin above 15mg/dL?**
    a. Phototherapy with 350-400 nm of violet light
    b. Phototherapy with 600-700 nm of red light
    c. Phototherapy with 500-570 nm of green light
    d. Phototherapy with 400-500 nm of blue light

**Answer Key**

1) a
2) c
3) b
4) b
5) c
6) b
7) c
8) d
9) d
10) b
11) c
12) c
13) a
14) d
15) b
16) c
17) a
18) d
19) b
20) d
