# Supplementary material for: Livogena: The Ikteros Curse—A Jaundice Narrative Card and Board Game for Medical Students
Source: MedEdPORTAL. 2024 Feb 6;20:11381. doi: 10.15766/mep_2374-8265.11381 (PMC10844581; doi:10.15766/mep_2374-8265.11381)
Supplement: Supplementary file 1 — Pre- and Posttest.docGame Instructions.docGame Story.docGame Video.mp4Game Board.jpgGame Cards.docxQuestions and Answer Key.docGame Activities.docPerceptions Questionnaire.docFeedback Questionnaire.doc [file mep_2374-8265.11381-s001.zip › B. Game Instructions.doc]

# Livogena: The Ikteros Curse

# Game Instructions

# Note: The game instructions should be shared with students online preferably a day before the game to clarify any doubts that they may have.

1. 150 Students shall be divided into 25 teams with 6 members in each. Each team shall have 3 sub-teams in them consisting of 2 players per sub-team. Hence, 3 sub-teams shall play with one game board.
2. Each team shall be allotted a faculty in charge who monitors and guides the players throughout the game.
3. The players shall decide which sub-team will start first, and the other sub-teams can follow. This order should not be changed throughout the game
4. The sub-teams shall take turns answering the questions by drawing any one of the playing cards one at a time from a deck of cards faced down.
5. Each team shall proceed on the game board with one coin. For every right answer that the sub-teams provide, the coin shall be moved on the game board in the forward direction.
6. Players can move forward on the game board as they give correct answers to the questions on the playing cards. With each correct answer, each team shall advance to the next box with the color on the board matching the color on the card.
7. The answered cards shall be kept separately and the unanswered cards for each round (after each sub-team has drawn a card) shall be collected by the faculty in charge. At the end of every round, the deck of cards shall be shuffled again by adding the unanswered cards of the previous round.
8. Players must complete the task for each character that they come across, to accomplish the goal related to that character as part of the mission. The advancement to the next same colored box is not possible without accomplishing the goal / by bypassing the character.
9. If a sub-team provides an incorrect answer or is unable to find the answer, the next sub-team will take the turn and proceed with drawing the card from the deck.
10. For the activities on the card, if the players are unable to find the answer, they can’t move further on the game board.
11. The first team to complete the game and save the King will be the winner.
12. Any discrepancies in the answers in the form of incorrect or multiple correct answers should be brought to the notice of the faculty in charge and chief organizer of the game.
13. All kinds of malpractice in the form of having other reference material, discussion of answers with other teams or prompts shall NOT be entertained
14. Here are three fundamental cards with 25 cards in each color, summing to 75 question cards and 25 special cards which include 5 Lucky Charm cards, 4 Hemocide cards, 4 Kernictears cards, and 3 Foucheter cards. Follow the instructions on the card as the game proceeds.

# Orientation to Game and Cards

- **75 Question Cards** will have questions for the players to answer
- **5 Lucky Charm Cards** which allow the players to advance one step forward without answering any questions – (it is not based on the color of the card to color on board)
- **4 HEMOCIDE Cards** which give the power to the playing team to make another team (choice of the playing team) lose their next turn.
- **4 KERNICTEARS Cards** which will cause the playing team to lose their next turn.
- **3 FOUCHETER Cards** for players to use in the current turn or save for the future to skip any of the questions. Note that these cards cannot be used to skip the activities for accessing the characters and accomplishing the goals of the mission.

**Question Cards**

- **Red, Yellow, and Violet**

**1. Lucky Charm Cards:**

**2. Hemocide Cards:**

**3. Kernictears Cards:**

**4. Foucheter Cards:**
