# Supplementary material for: Livogena: The Ikteros Curse—A Jaundice Narrative Card and Board Game for Medical Students
Source: MedEdPORTAL. 2024 Feb 6;20:11381. doi: 10.15766/mep_2374-8265.11381 (PMC10844581; doi:10.15766/mep_2374-8265.11381)
Supplement: Supplementary file 1 — Pre- and Posttest.docGame Instructions.docGame Story.docGame Video.mp4Game Board.jpgGame Cards.docxQuestions and Answer Key.docGame Activities.docPerceptions Questionnaire.docFeedback Questionnaire.doc [file mep_2374-8265.11381-s001.zip › C. Game Story.doc]

**Story of Livogena: “The Ikteros Curse”**

**Note:** This appendix need not be distributed to students. The story will be played as a video. This is just a written version of the story for reference. The game video should be played just before the start of the game. The time required for this is 5 minutes

“The Magic Whiteland Livogena consists of the Stellate Mainland and Island of Acina ruled by King Rubin and Queen Liverna, with newborn Prince Ehrlich as the only heir. Livogena is known for its crystals that are formed on the Scleroz Hills on the island of Acina. Only the winged unicorn - Spleendora, can cross the Bay of Bilis and reach the island. These crystals are believed to bring good fortune to the kingdom. The royal family and residents of Livogena worship the Sun God Helios to keep the crystals healthy. But with the evil intention of conquering Livogena, witch Verdina who lives in the Castle of Galls has poisoned the King and Queen with the Ikteros potion and has also abducted the newborn prince Ehrlich by casting a spell on the royal guards of the palace, Glucurons. Livogena and the crystals are now under the Ikteros curse. The crystals are losing their power and turning yellow! In addition, Galbinos - the evil monsters created by witch Verdina are causing havoc in the kingdom by killing innocent citizens and destroying their livelihood.

To save the royal family and the crystals, Sun-God Helios sends Angel Albumina who reveals that the antidote for Ikteros poison is the Hepasoteria drug found inside the Yakna Caves on the island of Acina. She seeks your help in her mission to save the royal family and the crystals before they lose their powers completely. Your mission is to find Spleendora who can take you to the island of Acina, win over the Galbinos, rescue Prince Ehrlich, find the antidote Hepasoteria, destroy witch Verdina and save the king and queen. But beware of the witch's evil spell which will hinder your progress in this mission. Don’t worry! it's not all bad, you'll meet Foucheter, the magic pearl 3 times during the mission. He will help you with the challenges that you face in this rescue mission and save the crystals. Good luck!”
