# Supplementary material for: Livogena: The Ikteros Curse—A Jaundice Narrative Card and Board Game for Medical Students
Source: MedEdPORTAL. 2024 Feb 6;20:11381. doi: 10.15766/mep_2374-8265.11381 (PMC10844581; doi:10.15766/mep_2374-8265.11381)
Supplement: Supplementary file 1 — Pre- and Posttest.docGame Instructions.docGame Story.docGame Video.mp4Game Board.jpgGame Cards.docxQuestions and Answer Key.docGame Activities.docPerceptions Questionnaire.docFeedback Questionnaire.doc [file mep_2374-8265.11381-s001.zip › G. Questions and Answer Key.doc]

LIVOGENA: “THE IKTEROS CURSE” QUESTIONS AND ANSWER KEYS

Note: This is the answer key for all the questions on the cards. Each facilitator will have a copy for this to cross-check the answers provided by students. This can later be distributed to students as educational material after the game.

(RED, VIOLET, AND YELLOW CARDS)

RED CARD ANSWER KEY

1. **Name the by-product of heme degradation whose deficiency causes microcytic hypochromic anaemia**

Iron

2. **Which reaction utilizes NADPH in the heme degradation pathway?**

Biliverdin to bilirubin by biliverdin reductase

3. **What are the effects of CO released during the degradation of heme to biliverdin?**

Vasodilation, anti-inflammatory action, and anti-proliferative action

4. **What is the end product of heme degradation in birds, amphibians, and rabbits?**

Biliverdin

5. **Mention the oxidation state of the mineral released as a by-product of heme degradation**

+2 (Fe + 2)

6. Name the transport protein for, lipophilic bilirubin formed in the reticulo-endothelial cells

Albumin

7. **Name any three drugs that can displace bilirubin from its transport protein.**

Aspirin, penicillin, dicoumarol, phenytoin, sulfa drugs, salicylates etc

8. **Name the cytosolic proteins to which bilirubin binds once it enters the liver cell**

Ligandin and Z protein

9. **Mention the other function of Ligandin**

Enzymatic activity involving detoxification using glutathione (GSH)

10. **Common treatment for neonatal hyperbilirubinemia when bilirubin levels rise above 15mg/dL**

Phototherapy with blue light 300-400 nm

11. **Which class of CNS depressants can be used to improve the symptoms of jaundice in Crigler-Najjar syndrome?**

Barbiturates

12. **How can mild jaundice be avoided in Gilbert’s disease without drug**

**supplementation?**

Avoidance of starvation

13. **What is the threshold value of bilirubin in blood above which it is known as hyperbilirubinemia?**

>1 mg/dL

14. **At what level does bilirubin diffuse into tissues producing yellow discoloration of sclera, conjunctiva, skin and mucous membranes?**

>2mg/dL

15. **What is the result of the Van den Berg test at pH 5 for hydrophilic	bilirubin?**

Direct positive (appearance of purple color immediately)

16. **What is the result of the Van den Berg test in blood with indirect bilirubin – 0.2-0.6 mg/dL and direct bilirubin 0-0.2 mg/dL?**

This is the normal level of bilirubin in blood which does not give a positive Van den Berg

17. **What is the reagent that is used in the test that measures urinary urobilinogen?**

Ehrlich test – para-dimethylamino benzaldehyde

18. **How is the liver function test in Dubin-Johnson syndrome?**

Liver function is normal in Dubin-Johnson syndrome

19. **Give the expected laboratory findings of blood-free bilirubin in pre-hepatic, hepatic and post-hepatic jaundice**

Pre – hepatic – increased Hepatic – increased

Post-hepatic – normal

20. **Give the expected laboratory findings of blood-conjugated bilirubin in pre-hepatic, hepatic and post-hepatic jaundice**

Pre–hepatic – normal

Hepatic – increased

Post-hepatic – increased

21. **Which isomer of coproporphyrin is increased in Dubin-Johnson syndrome?**

Coproporphyrin I

22. **Give the level of unconjugated bilirubin in blood in Crigler Najjar syndrome 1 & 2**

Unconjugated bilirubin level in blood exceeds 20mg/dL in Crigler-Najjar syndrome 1 and does not exceed 20mg/dL in Crigler-Najjar syndrome 2

23. **Sensorineural hearing loss is a serious and permanent effect in which complication of jaundice?**

Kernicterus – neonatal hyperbilirubinemia

24. **Conjugated hyperbilirubinemia, yellow discoloration of the bulbar conjunctiva, clay-colored stool and high levels of CA 19-9 are likely to suggest**

Pancreatic carcinoma (mostly head of the pancreas) obstructs the bile duct

25. **Yellowing of eyes and skin is also a common complication of which condition associated with increased accumulation of a substance required for heme synthesis, normal growth, and development.**

Hereditary Hemochromatosis (Iron overload)

VIOLET CARDS ANSWER KEY

1. **What is the metabolic defect in Black Liver Jaundice?**

Defective ATP-dependent organic anion transporter in biliary canaliculi resulting in elevated levels of conjugated bilirubin in blood.

2. **What are the non-pathological conditions that can cause mild jaundice in Gilbert’s disease?**

Stress and starvation

3. **Mention a cause for the non-hemolytic increase of unconjugated bilirubin in**

the blood

Crigler-Najjar syndrome

4. **High levels of estrogen derivative in fetal blood cause prolongation of jaundice in breastfed infants. True or false**

False. High levels of estrogen derivative in maternal blood cause prolongation of jaundice in breastfed infant

5. **Absence of bilirubinuria is seen in?**

Hemolytic jaundice

6. **What is the effect of bilirubin on brain mitochondria?**

Inhibits ATPase activity in brain mitochondria

7. **Enlarged lymph glands of porta hepatis may be a sign of obstruction leading to jaundice in which condition?**

Chronic hepatitis B

8. **What is the mode of inheritance of Dubin-Johnson syndrome?**

Autosomal recessive disease

9. **What is the drug of choice that is given to reduce pruritis in jaundice?**

Cholestyramine

10. **Administration of phenobarbital reduces blood level of bilirubin and is beneficial in which type of Crigler-Najjar syndrome?**

Type II

11. **What is the definitive treatment of Acalculous cholecystitis? – a form of cholecystitis that results in impaired gallbladder emptying leading to obstructive jaundice**

Cholecystectomy

12. **What are the dietary items that should be avoided in jaundice?**

Packed, canned, and smoked foods, Food with saturated and trans fats, Alcohol, Food with refined carbohydrates, Beef, and pork

13. **Give the normal value of serum Alkaline Phosphatase**

3-13AU/L or 8-125 U/L

14. **Give the normal value of SGOT & SGPT**

SGOT- 8-20 U/L

SGPT – Males – 13-35 U/L, Female - 10-30 U/L

15. **Name the other serum marker that raises during hemolysis and give its normal value**

LDH – Lactate Dehydrogenase

Normal value 100-200 U/L

16. **Give the normal level of total cholesterol in the blood**

150-200/dL

17. **What is MRCP in radiology?**

Magnetic Resonance Cholangiopancreatography – a special type of MRI that shows the liver, pancreas, bile ducts and gall bladder

18. **Give the expected laboratory findings of Urine- conjugated bilirubin in pre-hepatic, hepatic and post-hepatic jaundice**

Pre–hepatic – absent

Hepatic – present

post-hepatic – present

19. **Give the expected laboratory findings of Urine urobilinogen in pre-hepatic, hepatic and post-hepatic jaundice**

Pre – hepatic – increased

Hepatic – increased in the early phase, later decreased

Post-hepatic – absent

20. **Give the expected laboratory findings of fecal urobilinogen in pre-hepatic, hepatic and post-hepatic jaundice**

Pre–hepatic – increased

Hepatic – decreased

Post-hepatic – absent

21. **Is Delta bilirubin seen in the urine of patients with jaundice and normal glomerular function? Why?**

No. Delta bilirubin is albumin-bound conjugated bilirubin. Since albumin is not filtered through normal glomeruli, delta bilirubin is not seen in urine and thus cannot be detected in patients with jaundice and normal glomerular function

22. **What are the serological investigations that you would order to check for acute hepatitis B infection in a patient who presents with jaundice?**

HBsAg, anti-HBc, anti-HBs, IgM anti-HBc

23. **What is the first sign of recovery that is noticed in patients with obstructive jaundice?**

Reappearance of urobilinogen in urine

24. **Van den Berg test is indirectly positive, this suggests that the patient may be suffering from which type of jaundice?**

Hemolytic or pre-hepatic jaundice

25. **What is the first impaired activity in hepatocellular jaundice and what is the finding in the diazotized sulfanilic acid test?**

Excretion of bilirubin into bile. Thus, conjugated bilirubin levels increase in blood. This is seen as a direct positive in the diazotized sulfanilic acid test (Van den Berg test)

YELLOW CARDS ANSWER KEY

**1. Which reaction of monosaccharide is involved in the production of uronic acid utilized in the conjugation of insoluble compounds to soluble products?**

Oxidation of sugars – glucose is oxidized to glucuronic acid that combines with UDP to form UDP – glucuronic acid utilized in the conjugation reaction of bilirubin in the liver

**2. Name any three inhibitors of UDP glucuronyl transferase that interfere with the process of conjugation and may produce jaundice.**

Primaquine, novobiocin, chloramphenicol, androgens and pregnanediol.

**3. Which is the rate-limiting step in the catabolism of heme?**

Excretion of bilirubin into bile

**4. Name the ATP-dependent transporter of conjugated bilirubin that is responsible for the excretion of bilirubin into bile and give its location**

MOAT= Multispecific Organic Anion Transporter Location - plasma membrane of biliary canaliculi

**5. What is the function of intestinal bacteria on direct bilirubin?**

Intestinal bacteria **deconjugate** the direct bilirubin to UBG

**6. About 20% of UBG formed in the intestine is returned to the liver and a small fraction is also excreted in the urine as a result of**

Enterohepatic circulation

**7. Prolonged administration of antibiotics causes green-tinged feces, especially in children. Give reason**

Prolonged administration of antibiotics may decrease the intestinal flora and thus bilirubin is not reduced to bilinogens in the large intestine, it is oxidized by O2 to form biliverdin resulting in green-tinged feces.

**8. How can you assess the effectiveness of biliary drainage?**

Measurement of plasma delta bilirubin. An increased percentage of plasma delta bilirubin indicates the effectiveness of biliary drainage.

**9. What is the treatment of choice for a child born with severe hemolytic disease**

**/ Erythroblastosis fetalis?**

Exchange blood transfusion

**10. What is the route of administration of Rh immunoglobulins in a Rh-negative mother?**

Intramuscular or Intravenous

**11. What would be the suggested action in a TB patient who develops non-hemolytic hyperbilirubinemia due to intake of Rifampicin?**

As Rifampicin is a highly essential first-line anti-TB drug, it is suggested to continue Rifampicin with close monitoring of bilirubin as there is no hemolysis in this regard.

**12. Which test is used to measure the compounds that impart color to urine	and feces?**

Schlesinger’s test

**13. What is Hay’s test? And give the principle of the test**

Hay’s test is used to detect bile salts in urine. Bile salts lower the surface tension of the solution in which they are dissolved. When sulfur power is added, if bile salts are present in the urine, it sinks to the bottom while it floats on the surface of water when bile salts are absent

**14. Name the two tests that measure urinary conjugated bilirubin**

Fouchet’s and Gmelin’s tests

**15. How can you differentiate physiological and pathological jaundice with the time of onset of symptoms in a newborn infant?**

Physiological – 4 days after birth

Pathological – 1st or 2nd day after birth

**16. How does the specific gravity of urine get altered in hepatic jaundice?**

Specific gravity increases (hypersthenuria)

**17. Give the expected laboratory findings of serum Alkaline Phosphatase in pre-hepatic, hepatic and post-hepatic jaundice**

Pre–hepatic – normal

Hepatic – increased

Post-hepatic – very high

**18. Give the expected laboratory findings of Urine-bile salts in pre-hepatic, hepatic and post-hepatic jaundice**

Pre – hepatic – absent

Hepatic – absent

Post-hepatic – present

**19. In which type of jaundice Fouchet’s test results are negative**

Hemolytic or pre-hepatic

**20. What is the level of serum ALT, AST & ALP in Gilbert’s syndrome?**

ALT, AST & ALP – all are normal

**21. In chronic hepatocellular disorders, how is prothrombin time altered?**

Prothrombin time is often prolonged in chronic hepatocellular disorders that fail to respond to Parenteral administration of vitamin K.

**22. How does plasma albumin level change in hemolytic jaundice which results in increased production of unconjugated bilirubin?**

Plasma albumin level is normal. It does not change with the rise in bilirubin

**23. Mention other non-hepatic causes of rise in urine bilirubin (direct)**

Septicemia, hyperthyroidism

**24. Which vitamin intake may cause false-negative results in urine-conjugated bilirubin detection?**

Ascorbic acid (Vitamin C) intake

**25. The presence of covalently bound conjugated bilirubin in blood but the absence of conjugated bilirubin in urine may be due to which condition?**

**Prolonged cholestasis** where there is an increase in conjugated bilirubin in the blood (conjugated hyperbilirubinemia), but the predominant fraction of conjugated bilirubin is covalently bound to albumin called delta bilirubin which is not filtered by the glomerulus thus cannot be detected in urine
